# Effect and potential mechanism of oncometabolite succinate promotes distant metastasis of colorectal cancer by activating STAT3

**DOI:** 10.1186/s12876-024-03195-x

**Published:** 2024-03-14

**Authors:** Jiangnan Yu, Hong Yang, Lin Zhang, Suye Ran, Qing Shi, Pailan Peng, Qi Liu, Lingyu Song

**Affiliations:** 1 Department of Gastroenterology, Affiliated Hospital of Guizhou Medical University, Guiyang, 550004 China; 2 Department of Gastroenterology, Gui Zhou Hospital of the First Affiliated Hospital, Sun Yat-sen University, Guiyang, China

**Keywords:** Colorectal cancer, Succinate, STAT3, Migration and invasion, Epithelial-stromal transition

## Abstract

To investigate the effect of Oncometabolite succinate on colorectal cancer migration and invasion and to initially explore the underlying mechanism.Succinate acid detection kit detected the succinate content in tissues. The growth of colorectal cancer cells was measured by cck-8 assay, wound-healing migration assay and transwell migration and invasion assays, and then explored the level of epithelial-mesenchymal transition (EMT) and STAT3/ p-STAT3 expression by western blot analysis and quantitative real-time PCR for mRNA expression. We found that succinate levels were significantly higher in carcinoma tissues than paracancerous tissues. After succinate treatment, the colorectal cancer cell lines SW480 and HCT116 had enhanced migration and invasion, the expression of biomarkers of EMT was promoted, and significantly increased phosphorylation of STAT3. In vivo experiments also showed that succinate can increase p-STAT3 expression, promote the EMT process, and promote the distant metastasis of colorectal cancer in mice.Succinate promotes EMT through the activation of the transcription factor STAT3, thus promoting the migration and invasion of colorectal cancer.

**Supplementary Information:**

The online version contains supplementary material available at 10.1186/s12876-024-03195-x.

## Introduction

Colorectal cancer is a common malignant tumor of the digestive tract, and its incidence rate and mortality rate are increasing yearly. According to statistics, more than 930,000 patients die from colorectal cancer every year, ranking second in the cancer-related mortality rate [1]. The pathogenic factors of colorectal cancer are complex and closely related to genetics, poor lifestyle, environment and other factors [2]. At present, the main clinical treatments for colorectal cancer include surgery, chemotherapy, radiotherapy and targeted therapy. Although the 5-year survival rate of patients with early colorectal cancer undergoing radical resection exceeds 60%, most patients are already advanced or metastatic, resulting in a 5-year survival rate of 10% of patients with colorectal cancer, and the highly aggressive and metastatic nature of tumors are the key factors for their poor treatment effect and poor prognosis [3]. Therefore, studying the mechanisms of distant metastasis of colorectal cancer and effectively controlling cancer metastasis is a central issue in the treatment of colorectal cancer patients.

The tumor microenvironment is an important factor in cancer progression and metastasis [4]. Succinate is a metabolite produced by the microbiota and host and is traditionally recognized as one of the intermediate metabolites involved in multiple metabolic pathways, such as the tricarboxylic acid cycle and glutamic acid circulation. In recent years tumor formation and inflammation have been widely shown to play an important role, as cancer metabolite accumulation in some cancers can activate macrophages and polarization of tumor-related macrophages and enhance the migration of macrophages and the migration and invasion of cancer cells, thus promoting the development of tumors and metastasis. This factor is one of the most important components of the tumor microenvironment [5–6]. Previous studies have demonstrated the crucial involvement of succinate in distant metastasis, encompassing lung cancer, gastric cancer, and ovarian cancer. This phenomenon may be attributed to the succinate-induced polarization of macrophages towards tumor-associated macrophages, thereby facilitating cancer progression and metastasis while also conferring migratory potential on tumor cells for distant dissemination. Additionally, it promotes the expression of vascular endothelial growth factor by augmenting chemotactic movement and proliferation of vascular endothelial cells, ultimately fostering remote tumor metastasis [6–8].

Succinate has been reported to activate STAT3, an important nuclear transcription factor that regulates the inflammatory response [9]. As a signal transducer and activator of transcription, STAT3 is the key signaling molecule involved in the regulation of growth and malignant transformation, which are continuously activated in various human cancers, including colorectal cancer, and the activation of STAT3 is directly related to the prognosis of colorectal cancer [10]. This molecule can promote EMT to participate in tumor cell migration and invasion by upregulating MMP2, MMP9 and other factors and can directly activate VEGF and HIF-1α to promote tumor angiogenesis and other aspects to promote tumor metastasis. In addition, STAT3 can promote epithelial-mesenchymal transition (EMT) in colorectal cancer, thus promoting cancer metastasis [11–13]. Thus, the available evidence suggests an important link between succinate accumulation in the gut and inflammation and tumors. However, the role of succinate in distant metastasis of colorectal cancer remains unclear. This study aims to explore the effects of succinate on colorectal cancer migration and invasion, further explore the potential mechanisms, and provide new insights into the development of interventions to control colorectal cancer metastasis.

## Results

### Comparison of succinate levels in colorectal cancer tissues and pracancerous tissues

**Fig. 1:**
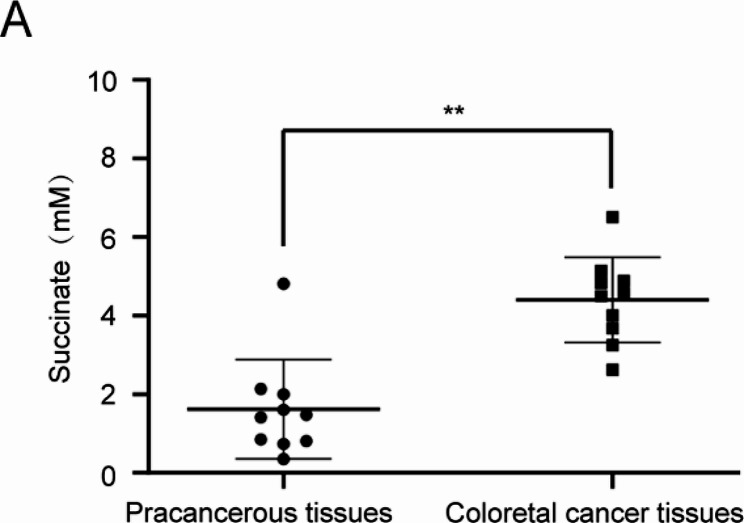
Succinate levels were significantly higher in human colorectal cancer tissues than in pracancerous tissues. **(A)** Succinate levels in colorectal cancer tissues were significantly higher than those in adjacent tissues compared to pracancerous tissues. Compared to pracancerous tissues, ^**^p < 0.01

A succinate acid detection kit was used to detect the succinate content in cancer tissues and pracancerous tissues. As shown in Fig. 1A, the level of succinate in colorectal cancer tissues was significantly higher than that in adjacent normal tissues. These findings suggest that colorectal cancer patients show marked variability, at least in succinate levels, possibly reflecting hallmarks of cancer development and progression.

### Succinate activates STAT3 and enhances migration and invasion of colorectal cancer cell lines

**Fig. 2:**
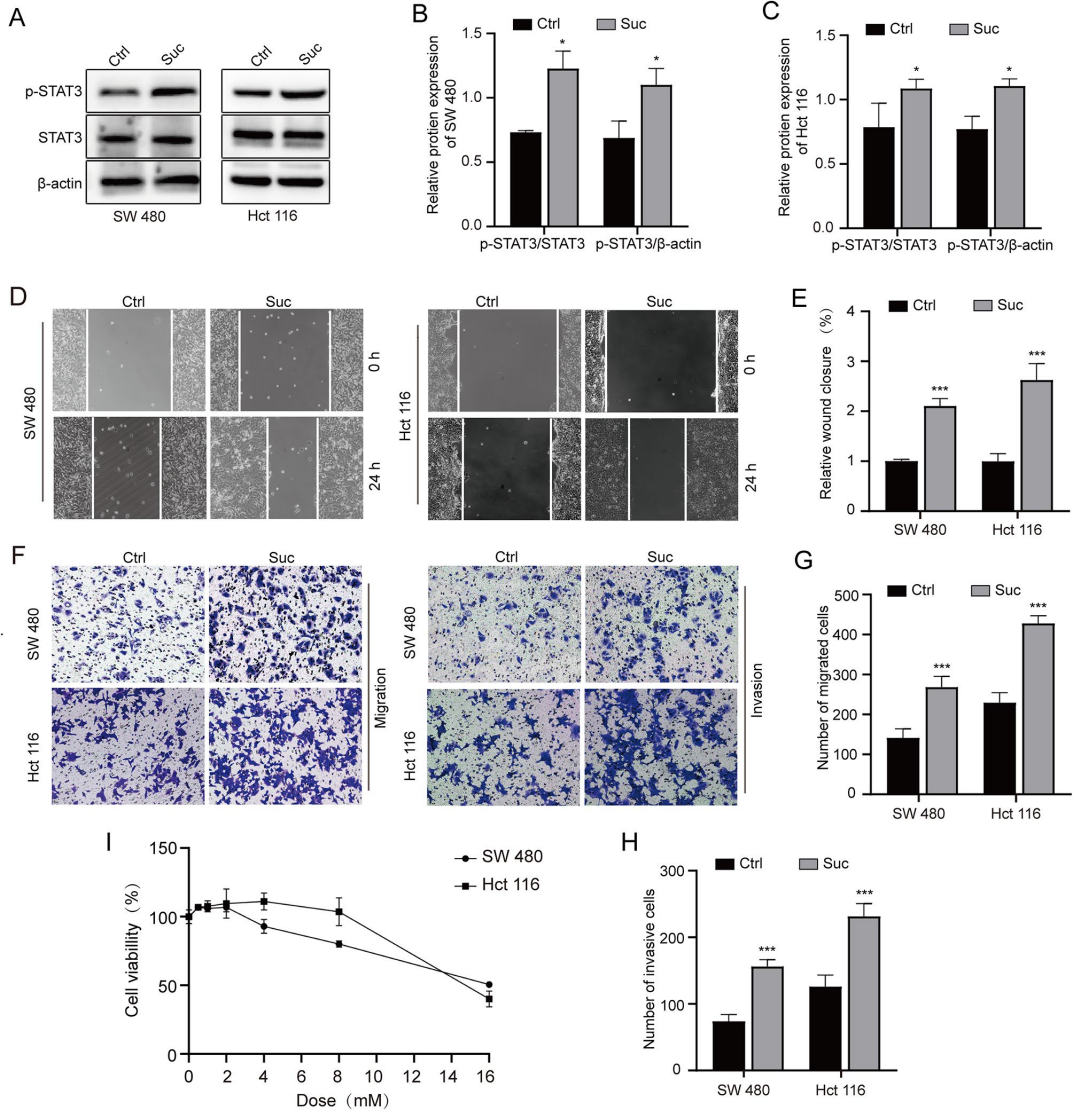
Succinate activates STAT3 and enhances migration and invasion of colorectal cancer cell lines. **(A-C)** The effect of succinate on STAT3 and p-STAT3 expression levels were assessed by western blot assays. **(D)** Wound healing migration assays to observe the effect on the colorectal cancer cell lines SW480 and HCT116; wound healing was photographed at 0 and 24 h. **(E)** Quantitative analysis of the wound-healing rate. **(F)** Cell migration and invasion assays were used to test the effect of succinate on the migration and invasion of colorectal cancer cells. (Magnification, 100x). **(G, H)** Quantitative analysis of transwell membrane cells. **(I)** CCK-8 cell proliferation assays to determine the effect of succinate on the activity of colorectal cancer cell lines (SW480, HCT116). ^**^p < 0.01, ^***^p < 0.001 compared to the controls

We explored the effect of succinate on colorectal cancer cell lines (SW480, HCT116). First, Our results showed that the expression of p-STAT3 was significantly increased (Figure 2A-C). To explore the effect of succinate on the migration and invasion of colorectal cancer cell lines (SW480, HCT116), we measured the migration and invasion of colorectal cancer cells by Wound-healing migration assay (Fig. 2D-E) and Cell migration and invasion assays (Fig. 2F-H). As shown in the figure, treatment with 2 mM succinate significantly increased the migration and invasion of colorectal cancer cell lines (SW480, HCT116) compared with those of the control group.We found that CCK-8 reagent had no significant effect on colorectal cancer cell lines (SW480, HCT116), while a high concentration of succinate had some inhibitory effect on cells, which may be related to drug toxicity (Fig. 2I).

### Inhibition of STAT3 phosphorylation by HO-3867 attenuates the ability of succinate to enhance migration and invasion of colorectal cancer cell

**Fig. 3:**
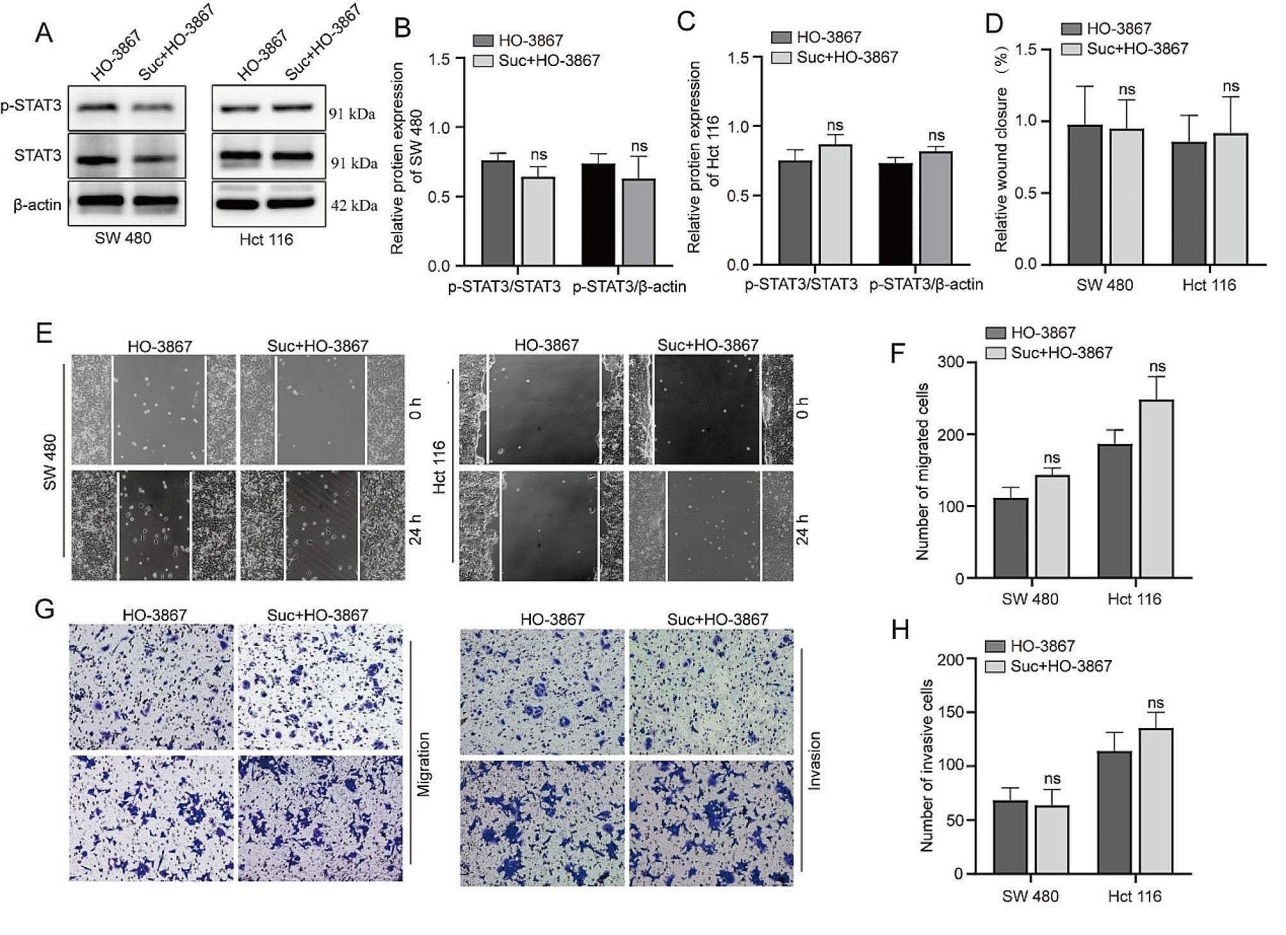
HO-3867 attenuates succinate-enhanced migration and invasion of colorectal cancer cells. **(A-C)** the effect of succinate on STAT3 and p-STAT3 expression levels were assessed by western blot assays after HO-3867 interfered with STAT3 phosphorylation. **(E)** wound healing migration assays to observe the effect on the colorectal cancer cell lines SW480 and HCT116; wound healing was photographed at 0 and 24 h. **(D)** Quantitative analysis of the wound-healing rate. **(G)** Cell migration and invasion assays were used to test the effect of succinate on the migration and invasion of colorectal cancer cells; (Magnification, 100x). **(F, H)** Quantitative analysis of transwell membrane cells. **(I)** CCK-8 cell proliferation assays to determine the effect of succinate on the activity of colorectal cancer cell lines. (It was performed under the same conditions and time as in Fig. 2, all samples derive from the same experiment and that gels/blots were processed in parallel)

Next, we sought to investigate the mechanism of pro-angiogenic function of succinate enhances the migration and invasion of colorectal cancer cell. HO-3867 is a well-known specific STAT3 phosphatase inhibitor, after we interfered with STAT3 phosphorylation by using HO-3867 (Figure 3A-C), the succinate-enhanced migration and invasion abilities was weakened (Figure 3D-H), *p*>0.05. These findings further suggest that succinate promotes colorectal cancer cell migration and invasion by activating STAT3 phosphorylation.

### Loss of epithelial features and acquisition of mesenchymal features are associated with succinate-activated STAT3

**Fig. 4:**
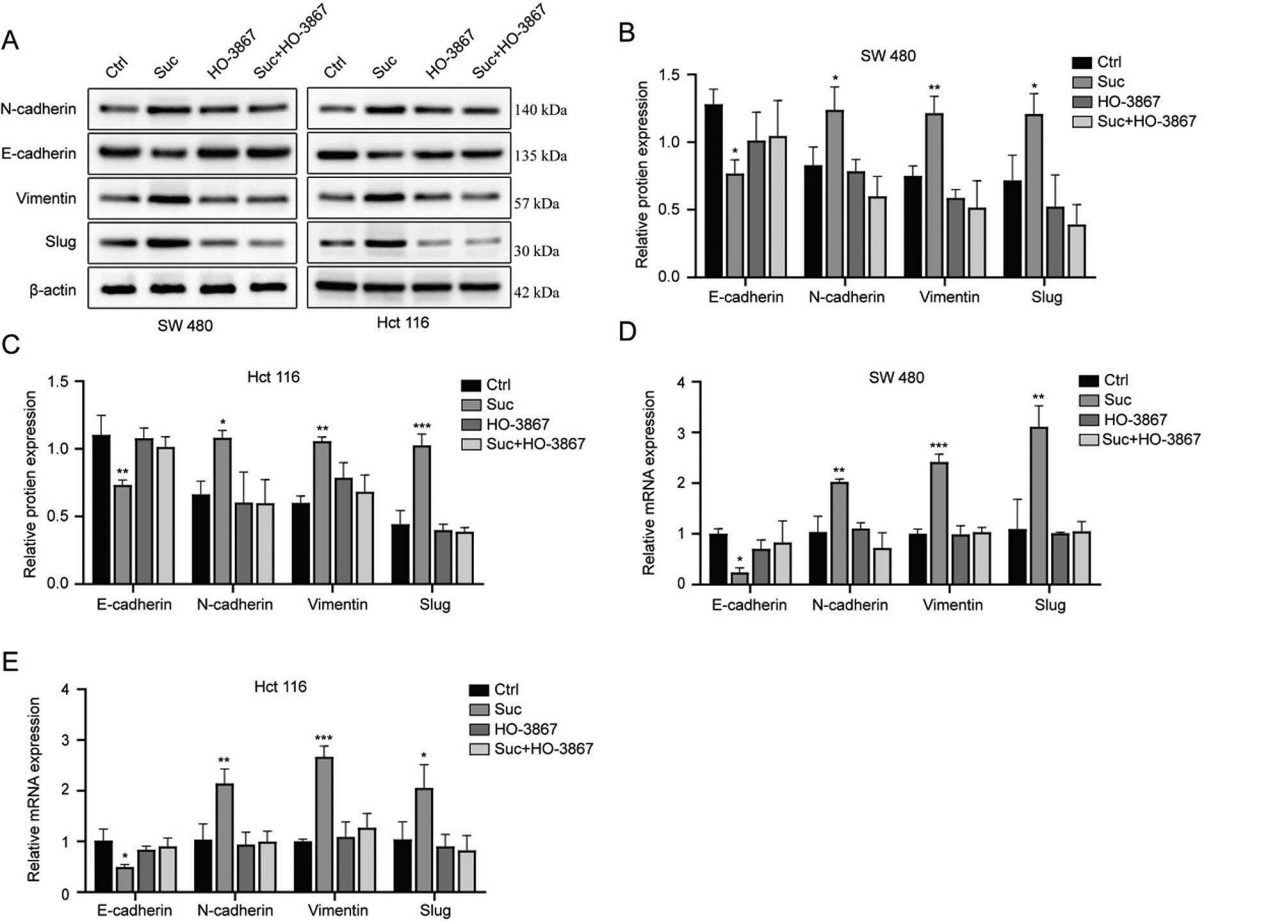
Succinate promotes the EMT process through activation of STAT3/p-STAT3. Treatment of the colorectal cancer cell lines SW480 and HCT116 for 48 h with 2 mM succinate and simultaneous inhibition of STAT3 phosphorylation by HO-3867 confirmed that succinate promotes the EMT process through activation of STAT3/p-STAT3. **(A-C)** Protein expression of the EMT markers E-cadherin, N-cadherin, Vimentin, and Slug was assessed by western blot analysis. **(D, E)** The mRNA expression of the EMT-related markers E-cadherin, N-cadherin, Vimentin and Slug was assessed by qRT‒PCR and analyzed by 2-ΔΔCT. ^*^p < 0.05, ^**^p < 0.01, ^***^p < 0.001 compared to the controls

One of the malignant behavioral characteristics of tumors is the spread of tumor cells, and the EMT process plays a key role in promoting the spread of colorectal cancer. Therefore, we further explored the possible mechanism by which succinate affects colorectal cancer cell motility by western blot analysis and qRT‒PCR. Our study showed that the expression level of the epithelial signature factor E-cadherin was decreased during EMT development, while the expression levels of the mesenchymal signature factors N-cadherin and Vimentin were increased after succinate treatment. Our results showed that the expression of p-STAT3, the upstream mediator of EMT, was significantly increased, and the effect of succinate in promoting the EMT process was weakened after inhibition of STAT3 phosphorylation by HO-3867 (Figure 4A-E).

### Succinate facilitates the EMT process through activation of STAT3/p-STAT3 in mice

**Fig. 5:**
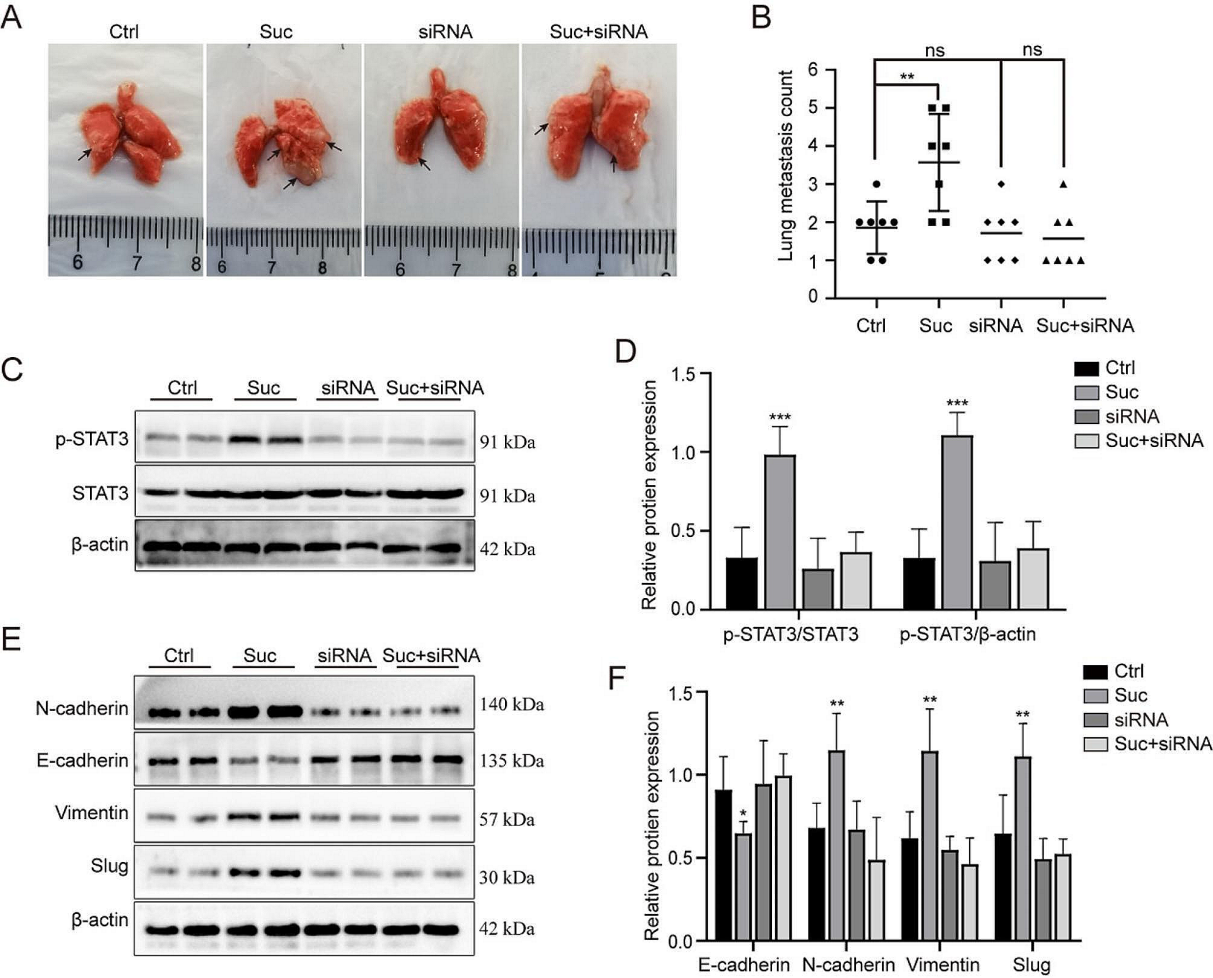
Succinate facilitates the EMT process through activation of STAT3/p-STAT3 in mice. **(A, B)** Representative lung metastatic nodules. **(C, D)** STAT3 and p-STAT3 expression was assessed by western blot analysis. **(E, F)** Protein expression of the EMT-related markers E-cadherin, N-cadherin, Vimentin, and Slug was assessed by western blot analysis. ^*^p < 0.05, ^**^p < 0.01, ^***^p < 0.001 compared to the controls

To verify whether succinate has a positive effect on colorectal cancer metastasis in vivo, we established a lung metastasis model by tail vein injection of HCT116 cells in nude mice. We divided the mice into control group, succinate treatment group, STAT3 inhibition group, STAT3 inhibition + succinate treatment group. After 8 weeks, the mice were killed, and lung tissues were collected. The lung metastasis model showed that the number of pulmonary surface metastatic nodules was significantly higher in the succinate-treated group than in the control group (Fig. 5A,B). and then animal proteins were extracted from lung tissue for western blot analysis, which further showed that succinate could increase the phosphorylation of STAT3 (Fig. 5C,D) and promote EMT in tumor tissue (Fig. 5E,F). In addition, the impact of succinate on EMT was nullified upon inhibition of STAT3 phosphorylation.These results are consistent with our in vitro experimental findings.

## Discussion

Colorectal cancer is a common cancer worldwide, and distant metastasis is a key factor in its death, so it is crucial to study the mechanism of distant metastasis of colorectal cancer and find new treatment options. Succinate was found to be a tumor source of a new class of cancer progression factors [14–15]. Wu et al. [6] found that the serum succinic acid content of lung cancer patients increased, and succinic acid secreted by tumor cells belongs to a new class of cancer progression factors, which enter the extracellular environment, bind to the specific membrane receptor GPR91, and induce the polarization of macrophages into tumor-associated macrophages. The signals produced by these macrophages control the functional phenotypes of various non-cancer cells in their vicinity, which in turn promote cancer growth and metastasis [16]. Ting Jiang et al. [17] urine succinate concentration is significantly increased in ovarian cancer patients, and the accumulation of succinate in ovarian cancer can promote energy production and contribute to cell proliferation and migration by maintaining the tricarboxylic acid cycle [7]. Mu et al. [8] found that the accumulation of succinate was significantly increased in gastric cancer tissues, which may be related to the activation of signal transducer and activator of transcription STAT3 and extracellular regulated kinase ERK1/2 by succinate, which up-regulate the expression of vascular endothelial growth factor through its receptor GPR91 in a HIF-1α independent mechanism. Furthermore, it increases the chemotactic movement and proliferation of vascular endothelial cells. All these studies suggest that succinate plays an important role in the distant metastasis of tumors.However, there is no evidence for a role of succinate in colorectal cancer. This study found that succinate could significantly enhance the metastasis and invasion of HCT116 and SW480 cells and enhance p-STAT3 expression and the EMT process.

In the tumor microenvironment, cancer cells release signaling molecules to activate their own oncogenic signals to change the surrounding cells and the environment to promote tumor progression. Alterations in cell metabolism have been recognized as markers of cancer cells, providing new potential targets in terms of tumor therapy. Specific metabolites can maintain normal physiological processes, and abnormal metabolite accumulation plays a key role in promoting the progression of the disease. The inflammatory microenvironment of tumors is composed of a large number of cytokines and various proteolytic enzymes involved in the inflammatory response, which change the cellular environment and induce the inflammatory cancer transformation of normal cells [18–19]. The role of succinate in addition to metabolism is gradually being evaluated. This molecule also acts as a signal of inflammation to promote macrophage inflammatory factor IL-1 β production and increase fibroblast IL-6 production by stabilizing HIF-1α [20–21]. Increased succinate was found in both the feces of mice with IBD and humans and was shown to be associated with disease activity [22]. Metagenomic studies of the gut microbiome of IBD patients found significantly lower levels of specific succinate-consuming bacterial strains [23–24]. All of these findings suggest that succinate plays an important role in the intestinal inflammatory response, and the inflammatory response is an important factor in the development of colorectal cancer. However, the role of succinate in colorectal cancer has not been proven. We detected the levels of succinate in cancer tissues and adjacent tissues of colorectal cancer patients undergoing radical resection and found that the levels of succinate in colorectal cancer tissues were significantly higher than those in adjacent normal tissues, further confirming that succinate may play an important role in the development of colorectal cancer. Unfortunately, we did not further determine whether succinate accumulates in the cytosol or extracellular space.

The STAT family includes multiple activators of signal transduction and transcription, in which STAT3 is involved in multiple biological processes, including cell survival, proliferation, differentiation, tumorigenesis and angiogenesis [25–26]. Under certain stimulation conditions, the STAT3 pathway is hyperactivated by high levels of IL-6 through direct phosphorylation of tyrosine and serine residues [27]. STAT3 activation has important effects on both cell growth and apoptosis and the regulation of the cell cycle. Multiple studies have confirmed that STAT3 is hyperactivated in most human cancers, is activated in many solid and hematological tumors and is associated with poor clinical outcomes [28]. Studies have reported that STAT3 overexpression plays a crucial pathogenic role in the development, progression and metastasis of colorectal cancer [29]. As an upstream mediator of EMT, STAT3 can upregulate EMT expression in brain tumors, lung cancer and gastrointestinal cancer to mediate tumor metastasis and plays an important role in the development of various tumors [30]. During EMT, epithelial-derived tumor cells lose cell polarity, intercellular junctions become loose, and the cytoskeleton reorganizes, resulting in decreased adhesion of tumor cells, which significantly enhances the migration and invasion of cells. During the development of EMT, the expression level of E-cadherin (E-cadherin), which is an epithelial indicator, decreased, and the expression of mesenchymal cell characteristic molecules (such as N-cadherin and Vimentin) increased, which is considered to be a key step in the migration and metastasis of tumor cells [31]. To verify the effect of succinate on colorectal cancer migration and invasion, we confirmed that the migration, invasion and EMT processes were strengthened after succinate treatment. In addition, we used a STAT3 inhibitor to study succinate-driven STAT3 phosphorylation to affect tumor migration, and our results showed that when the colorectal cancer cell lines SW480 and HCT116 were treated with the STAT3 inhibitor HO-3867, the migration, invasion and EMT processes were weakened. In vivo experiments further confirmed that succinate can increase STAT3 phosphorylation expression and strengthen the EMT process, consistent with in vivo experiments, but unfortunately, we did not further verify whether succinate still promotes tumor metastasis after interfering with STAT3 phosphorylation in vivo.

In conclusion, succinate promotes the metastasis and invasion of colorectal cancer, and when cancer cells are specifically inhibited by a STAT3 inhibitor, the effect of succinate in promoting cancer cell metastasis and invasion is weakened. This process may be associated with succinate activation of STAT3, which upregulates EMT-related expression and subsequently promotes distant metastasis in colorectal cancer. These findings have potential clinical implications and provide an important reference for the treatment and prognosis of colorectal cancer patients. However, the specific mechanism of abnormal expression of succinate in tumor tissues needs to be further explored.

## Materials and methods

### Human tissue

From September 2021 to March 2022, 10 fresh colorectal cancer tissues and paired adjacent normal mucosa were collected from colorectal cancer patients undergoing radical resection at the Affiliated Hospital of Guizhou Medical University and immediately cryopreserved in liquid nitrogen. Diagnosis of all specimens was confirmed by histopathology. Ethics approval and consent to participate were approved by the Ethics Committee of the Affiliated Hospital of Guizhou Medical University.(Ethical batch number: 2020 Lun Shen No.031). The basic patients information are listed in Table 1.

**Table 1:** Characteristics of the participants

Patient.No	Sex	Age	BMI(kg/m^2^)	Sites	Differentiation	Tumor-stage	Lymphovascular invasion
1	F	34	21.9	Sigmoid	Moderate	T2N0M0	No
2	F	45	18.4	Sigmoid	Moderate	T3N0M0	No
3	M	69	23.1	Rectal	Moderate	T3N0M0	No
4	F	84	21.4	Colon	Moderate	T3N1M0	No
5	M	47	28.7	Rectal	Moderate	T3N0M0	Yes
6	F	54	23.4	Sigmoid	Moderate/Poor	T3N0M0	No
7	M	69	20.2	Rectal	Undifferentiated	T4N1M0	No
8	M	79	20.8	Rectal	Moderate	T2N0M1	No
9	M	75	28.0	Rectal	Well/moderate	T3N1M0	No
10	M	66	21.5	Colon	Moderate	T3N2M0	No

### Animals

Five-week-old male athymic BALB/c nude mice (Beijing Weitong Lihua Experimental Animal Technology Co., Ltd., China) were bred under standard pathogen-free conditions in the Experimental Center of the Affiliated Hospital of Guizhou Medical University, with alternating light and dark cycles of 12 h/12 h. Mice were randomly divided into 4 groups (*n* = 7 per group), control group and succinate treatment group were injected negative control lentivirus (GeneChem, China) of HCT116 cells (2*10^^6^/0.2 ml) into the tail vein, STAT3 inhibition group and STAT3 inhibition + succinate treatment group were injected lentivirus expressing STAT3 siRNA (GeneChem, China) of HCT116 cells (2*10^^6^/0.2 ml)into the tail vein to construct colorectal cancer metastasis model, and fed pure water and 2.5% succinate (Shanghai Aladdin Biochemical Technology Co., Ltd., China) respectively. After eight weeks, the mice were sacrificed by abdominal anesthesia with 100 mg/Kg of 3% sodium pentobarbital. After the complete unconsciousness of the mice was confirmed, the lung tissue was harvested, frozen in liquid nitrogen, and stored at -80 °C. The study is reported in accordance with ARRIVE guidelines, This study was approved by the Ethics Committee of Guizhou Medical University(Ethical batch number: NO.2,000,638).

### Cell culture

The human colorectal cancer cell lines HCT116 and SW480 (Obtained from Zhong Qiao Xin Zhou Biotechnology Co., Ltd., China) were cultured in DMEM (Gibco BRL, Grand Island, NY) complete medium containing 10%, FBS and 1% penicillin/streptomycin (Zhong Qiao Xin Zhou Biotechnology Co., Ltd., China) and incubated in an incubator containing 37 °C and 5% CO2. Fresh medium was exchanged every 2 days, and when the cell density reached 80–90%, the cells were digested with trypsin solution for subculture or plate laying. HO-3867 (10 µmol/l, MedChemExpress LLC, China) is a STAT3 inhibitor used to inhibit the expression of STAT3 [32].

### Succinate level measurement

The succinate level measurement was determined by a succinate detection kit (Sigma-Aldrich, USA) according to the instructions of the test box; 100 mg of tissue and sterile enzyme-free water were placed on ice, insoluble material was removed at 5 min at 14,000 rpm and the supernatant was collected to obtain samples. Then, 20 µl of sample was added to the 96-well plate, the working solution and standard were added, the samples were mixed with light shaking and incubated at room temperature for 30 min, absorbance was measured at 570 nm, and replicate wells were used for each sample.

### Cell counting Kit-8 (CCK-8) assay

Cell viability was assessed using the CCK-8 assay (Dojindo Laboratories, Japan). HCT116 and SW480 cells were seeded in 96-well plates (1*10^4 cells/well). After cell attachment, 10 µl of CCK-8 reagent was added to 96-well plates, and the cells were incubated at 37 °C for 2 h before cell viability was measured using a multifunctional microplate reader (Molecular Devices). Then, the absorbance was measured at 450 nm.

### Wound-healing migration assay

Wound-healing assays were used to determine cell migration. SW480 cells (3*10^^5^ cells/well) were seeded in 6-well plates. After 80% confluence was reached, a straight line was drawn on the cell monolayer using a 200 µl pipette tip. After the cell debris were washed three times with PBS, the cells were cultured with serum-free DMEM at 37 °C for 24 h. Images of migrating cells were captured at 0 and 24 h under an optical microscope (magnification, 100x; Ci-E, Nikon). Finally, the data were analyzed by ImageJ.

### Transwell migration and invasion assay

For detection of cell invasion, Transwell plates (Labselec, China) with 8-µm wells were used, with Matrigel in the Transwell upper chamber. HCT116 and SW480 cells (4*10^4 cells/well) were seeded with 200 µl of cell suspension in the upper chamber, and 600 µl of DMEM with 10% FBS was added to the lower chamber. After 48 h of incubation at 37 °C with the beads, the cells were fixed with 4% polyformaldehyde at 37 °C for 15 min and stained with 0.1% crystal violet at 37 °C for 10 min. The cells were then observed and counted under an optical microscope (magnification, 100x; Ci-E, Nikon). For cell migration analysis, no Matrigel step followed the invasion assay. Finally, the data were analyzed by ImageJ.

### Quantitative real-time PCR for mRNA expression analyses (qRT‒PCR)

Total RNA was extracted from six-well plates of the colorectal cancer cell lines SW480 and HCT116 treated with succinate or inhibitor for 48 h. The cDNA was synthesized using the cDNA Reverse transcription kit (TaKaRa, Japan). qRT‒PCR was performed using SYBR Premix Ex Taq (TaKaRa, Japan) and then analyzed by a StepOne real-time PCR system (Applied Biosystem) under the following reaction conditions: 5℃ predenaturation for 2 min; 95℃ denaturation for 10 s, 60℃ annealing for 30 s, and 72℃ extension for 15 s. Relative gene expression was calculated using the 2- ΔΔCt method, where a higher 2- ΔΔCt value reflects higher expression. The primer sequences are listed in Table 2.

**Table 2:** Primers used for qRT-PCR analysis

Gene	Forward primer sequence(5’-3’)	Reverse primer sequence(5’-3’)
E-adherin	CGAGAGCTACACGTTCACGG	GGGTGTCGAGGGAAAAATAGG
N-adherin	TTTGATGGAGGTCTCCTAACACC	ACGTTTAACACGTTGGAAATGTG
Vimentin	GCCGTTGAAGCTGCTAACTA	CCAGAGGGAGTGAATCCAGATTA
Slug	AACTACAGCGAACTGGACACACATAC	CGTGGAATGGAGCAGCGGTAG
GAPDH	CTCCTCCTGTTCGACAGTCAGC	CCCAATACGACCAAATCCGTT

### Western blot analysis

Lung tissue and colorectal cancer cells were harvested with RIPA buffer (Beyotime Biotechnology, China) and protease inhibitors (Roche Applied Science, USA) at 4 °C for 30 min, and a BCA kit (Beyotime Biotechnology, China) was used to detect the protein concentration. The samples were denatured in a 100 °C water bath, ultrasonicated, and stored at -80 °C. The extracted protein samples were loaded on an SDS-polyacrylamide gel for electrophoresis and then transferred to a PVDF membrane. The membrane was then incubated with the corresponding primary antibodies against E-cadherin (Cell Signaling, 1:1000), N-cadherin (Cell Signaling, 1:1000), Vimentin (Cell Signaling, 1:1000), Slug (Cell Signaling, 1:1000), STAT3 (Wanleibio, 1:300), and p-STAT3 (Wanleibio, 1:1000) overnight at 4 °C. Blots were cut prior to hybridisation with antibodies during blotting,The HRP-conjugated secondary antibody was used to visualize protein expression (ZSGB-BIO). Detection was performed by the GENEsys system, and protein bands were analyzed by ImageJ.

### Statistical analysis

All experiments were repeated in triplicate. The Shapiro-Wilk method was employed to assess the normality of continuous variables. If the data followed a normal distribution, statistical description was presented as mean ±  SEM. Independent sample t-test or one-way analysis of variance was used for comparing differences between groups, and multiple comparisons were conducted using the Bonferroni method. In cases where the data did not follow a normal distribution, statistical description was provided as median (lower quartile Q1, upper quartile Q3). Non-parametric Mann-Whitney U test or Kruskal-Wallis H test was utilized for comparing differences, and multiple comparisons were performed using Dunn’s test. Statistical analysis was carried out using SPSS 26.0 and GraphPad Prism 9. The significance level α = 0.05, with *P* < 0.05 considered statistically significant.

## Electronic supplementary material

Below is the link to the electronic supplementary material.

Supplementary Material 1

Supplementary Material 2

Supplementary Material 3

## Data Availability

The datasets used or analyzed during the current study are available from the corresponding author on reasonable request.
