# Correction: Effect and potential mechanism of oncometabolite succinate promotes distant metastasis of colorectal cancer by activating STAT3

**DOI:** 10.1186/s12876-024-03396-4

**Published:** 2024-09-03

**Authors:** Jiangnan Yu, Hong Yang, Lin Zhang, Suye Ran, Qing Shi, Pailan Peng, Qi Liu, Lingyu Song

**Affiliations:** 1 Department of Gastroenterology, Affiliated Hospital of Guizhou Medical University, Guiyang, 550004 China; 2 Department of Gastroenterology, Gui Zhou Hospital of the First Affiliated Hospital,Sun Yat-sen University, Guiyang, China

**Correction: BMC Gastroenterol 24, 106 (2024)**

10.1186/s12876-024-03195-x

Following publication of the original article the authors reported an error in affiliation 2. In the original publication it was originally given as: Department of Gastroenterology, The First Afliated Hospital of Sun Yat-sen Medical University Guizhou Branch, Guiyang, China

The correct affiliation 2 is: Department of Gastroenterology, Gui Zhou Hospital of the First Affiliated Hospital, Sun Yat-sen University, Guiyang, China.

The original article has been updated.

