# Correction: Effect and potential mechanism of oncometabolite succinate promotes distant metastasis of colorectal cancer by activating STAT3

**DOI:** 10.1186/s12876-024-03478-3

**Published:** 2024-11-05

**Authors:** Jiangnan Yu, Hong Yang, Lin Zhang, Suye Ran, Qing Shi, Pailan Peng, Qi Liu, Lingyu Song

**Affiliations:** 1 Department of Gastroenterology, Affiliated Hospital of Guizhou Medical University, Guiyang, 550004 China; 2 Department of Gastroenterology, Gui Zhou Hospital of the First Affiliated Hospital, Sun Yat-Sen University, Guiyang, China

**Correction** **:** **BMC Gastroenterol 24, 106 (2024)**

**https://doi.org/10.1186/s12876-024-03195-x**

Following publication of the original article [1] it was reported that there was an error with Fig. 2, specifically panel 2F. The image used for Fig. 2F was a repetition of Fig. 3G.

The incorrect Fig. 2 is shown below:


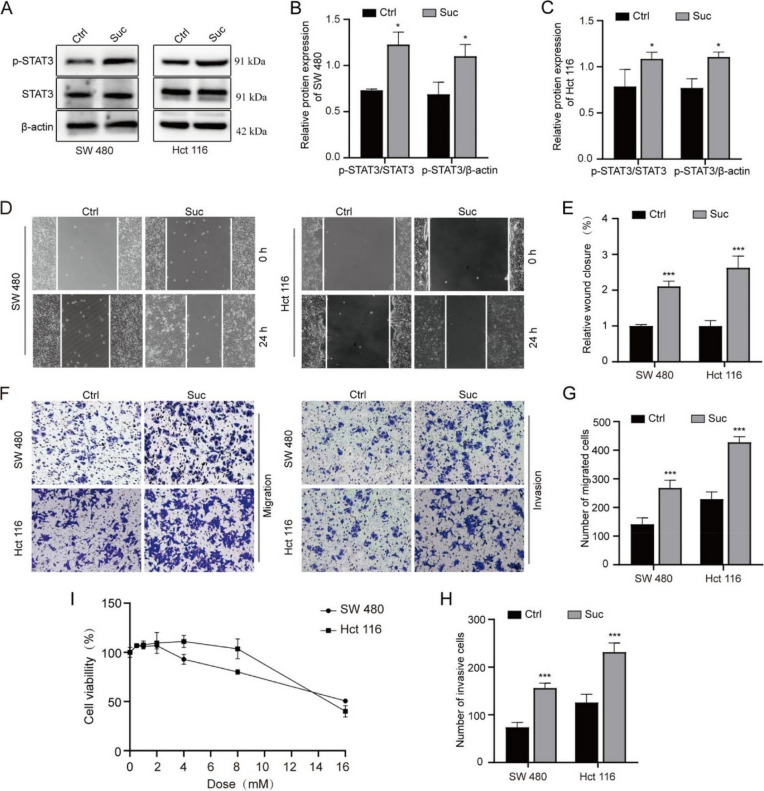


The correct Fig. 2 is:


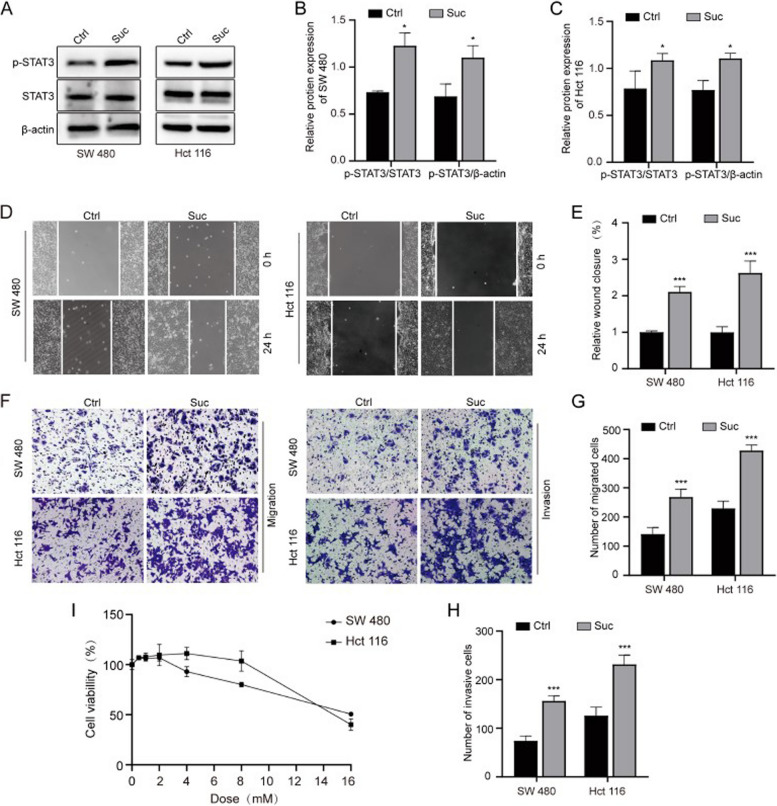


The original article has been updated.
