# Supplementary material for: Effect and potential mechanism of oncometabolite succinate promotes distant metastasis of colorectal cancer by activating STAT3
Source: BMC Gastroenterol. 2024 Mar 14;24:106. doi: 10.1186/s12876-024-03195-x (PMC10938789; doi:10.1186/s12876-024-03195-x)

HCT 116

We performed this simultaneously in 4 groups(From left to right is：ctrl、Suc、HO+3867、Suc+HO-3867). In order to write the logic of the article, we separately describe in Figure 2 and Figure 3 in the article. Since some of the samples were in close proximity to the gel, we had to crop the bands, and we guarantee that the samples derive from the same experiment and that gels/blots were processed in parallel.

Hct 116 β-actin
Figure4A

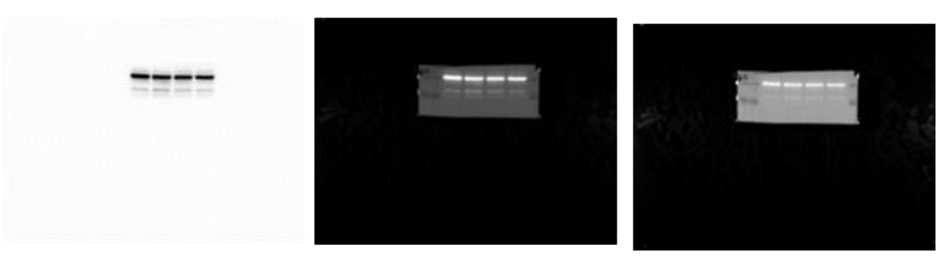

Hct 116 β-actin
Figure2A and Figure3A

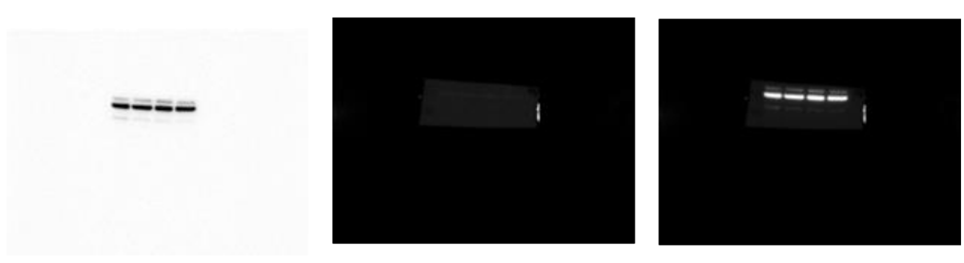

Hct 116 β-actin
replicates

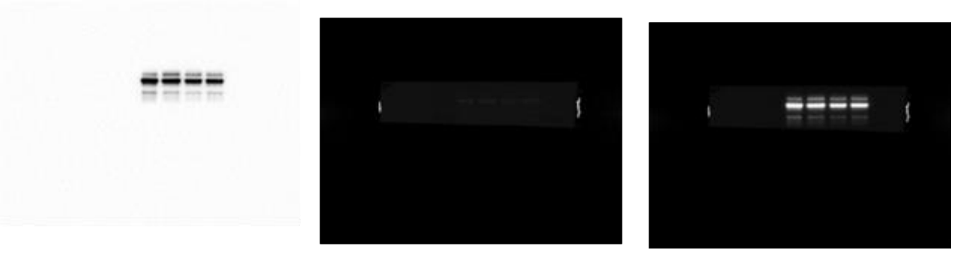

Hct 116 STAT3
replicates

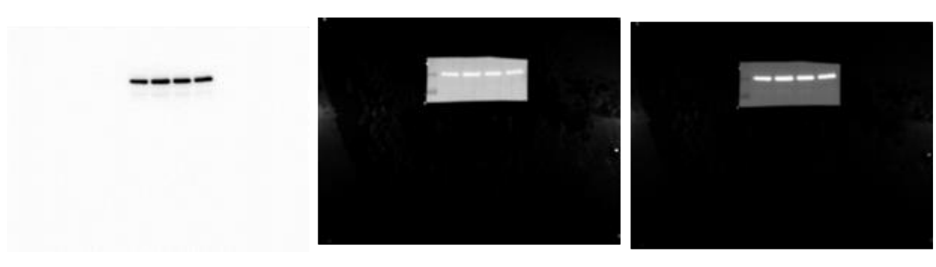

Hct 116 STAT3
Figure2A and Figure3A

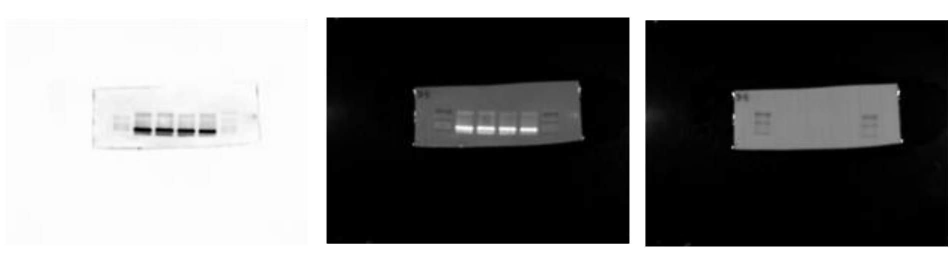

Hct 116 STAT3
replicates

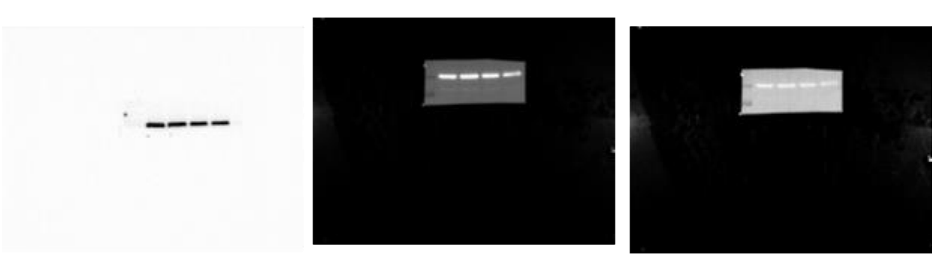

Hct 116 P-STAT3
Figure3A

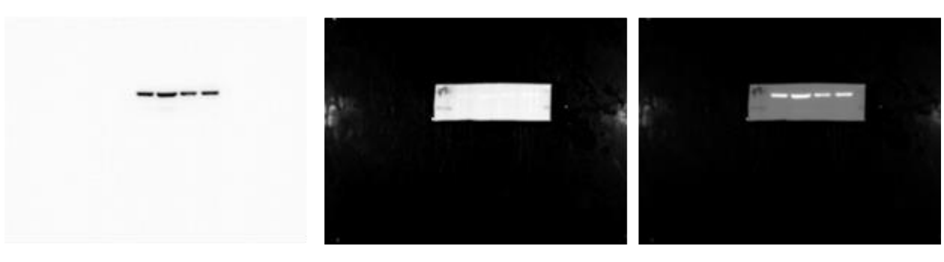

Hct 116 P-STAT3
Figure2 A

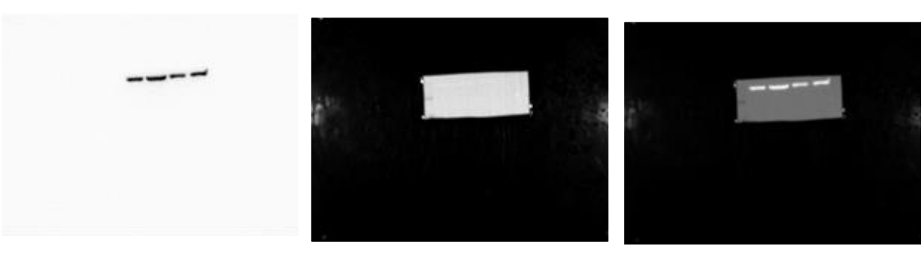

Hct 116 P-STAT3
replicates

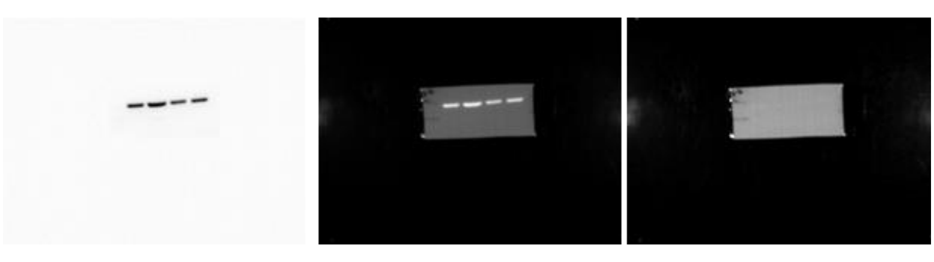

Hct 116 Vimentin
Figure4A

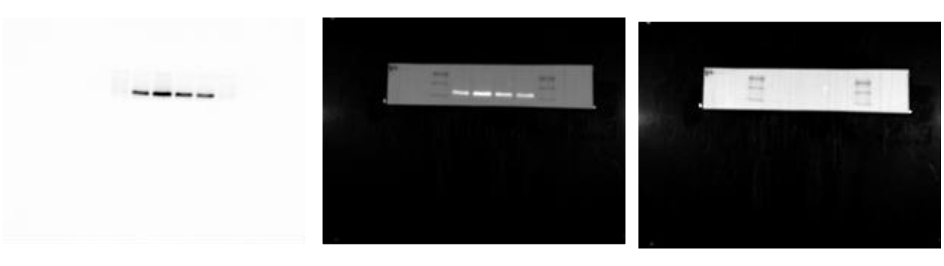

Hct 116 Vimentin
replicates

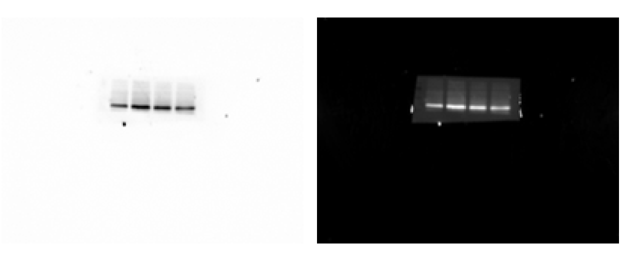

Hct 116 Vimentin
replicates

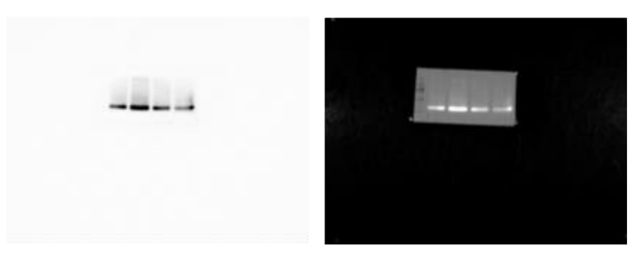

Hct 116 E-cadherin
Figure4A

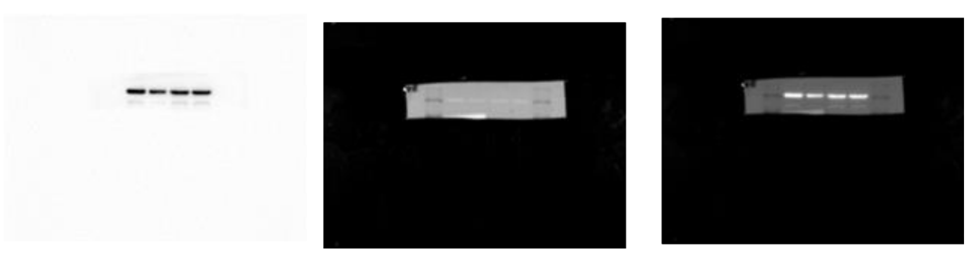

Hct 116 E-cadherin
replicates

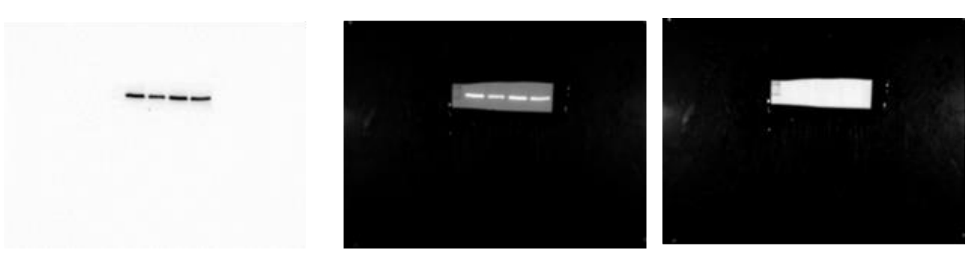

Hct 116 E-cadherin
replicates

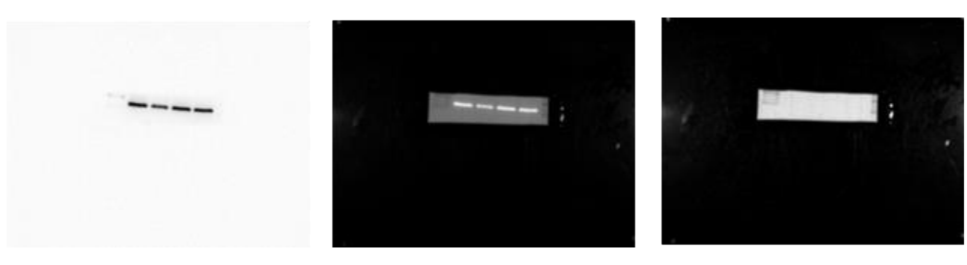

Hct 116 N-cadherin
replicates

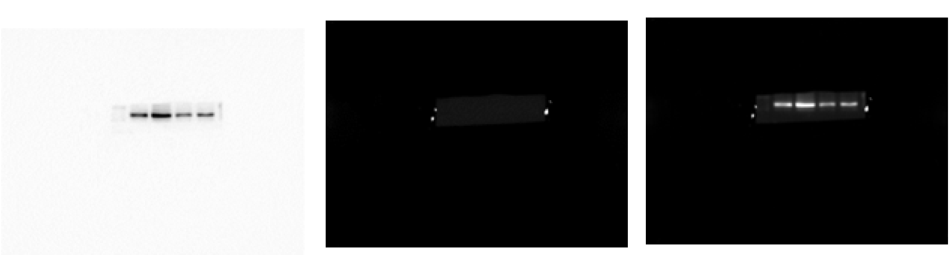

Hct 116 N-cadherin
replicates

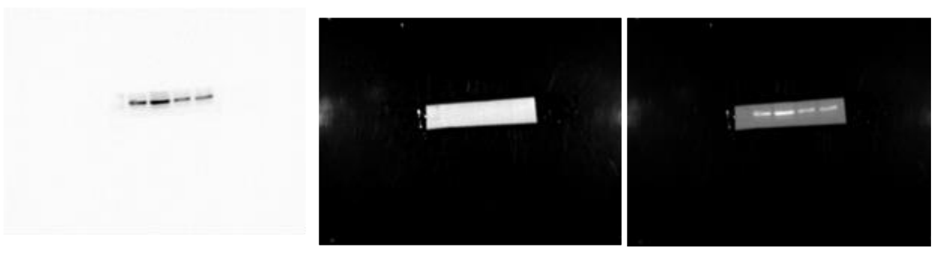

Hct 116
N-cadherin
Figure4A

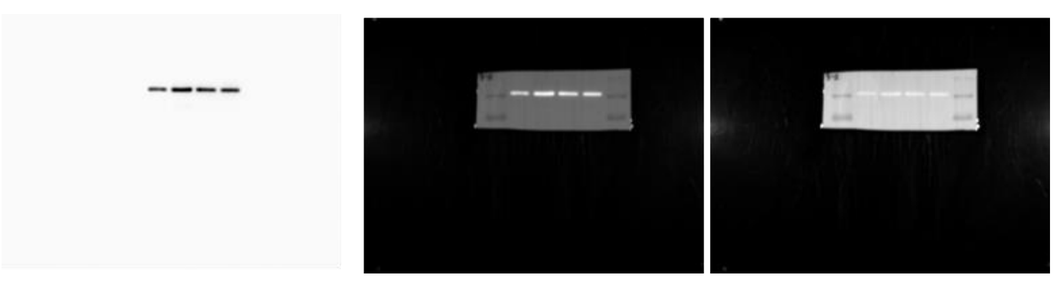

Hct 116 Slug
Figure4A

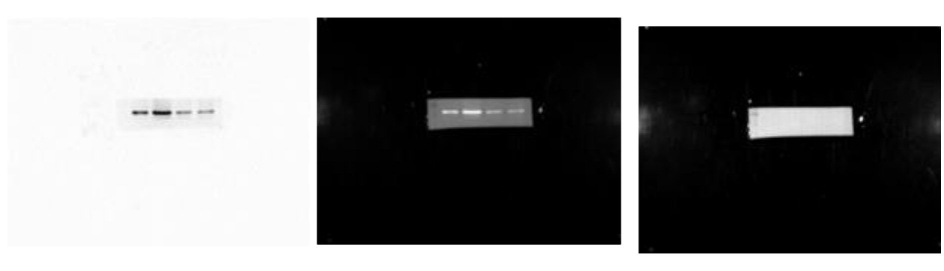

Hct 116 Slug
replicates

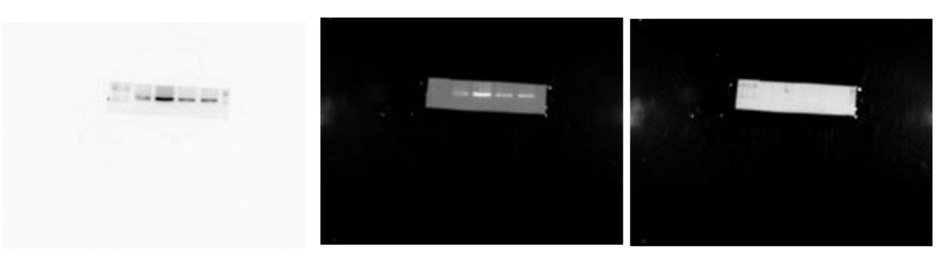

Hct 116 Slug
replicates

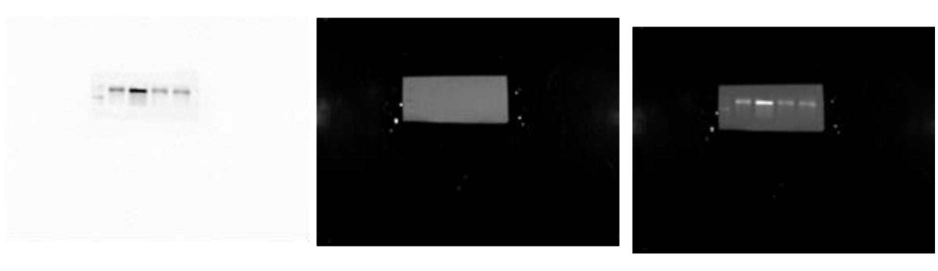

Supplement: Supplementary file 1 — Supplementary Material 1 [file 12876_2024_3195_MOESM1_ESM.pdf]
