# Supplementary material for: Effect and potential mechanism of oncometabolite succinate promotes distant metastasis of colorectal cancer by activating STAT3
Source: BMC Gastroenterol. 2024 Mar 14;24:106. doi: 10.1186/s12876-024-03195-x (PMC10938789; doi:10.1186/s12876-024-03195-x)

We performed this simultaneously in 4 groups(From left to right is：ctrl、Suc、HO+3867、Suc+HO-3867), In order to write the logic of the article, we separately describe in Figure 2 and Figure 3 in the article. Since some of the samples were in close proximity to the gel, we had to crop the bands, and we guarantee that the samples derive from the same experiment and that gels/blots were processed in parallel.

SW 480
β -actin
replicates

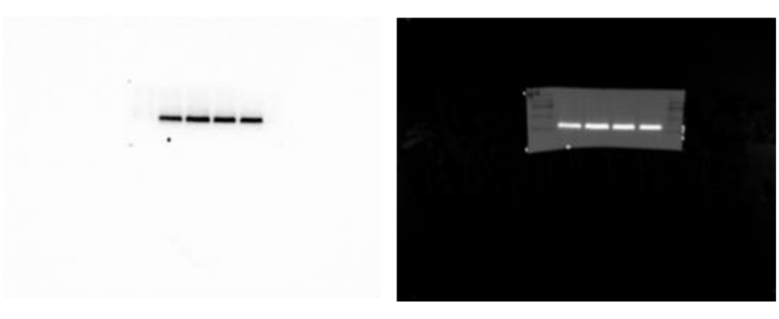

SW 480 β -actin
Figure4A

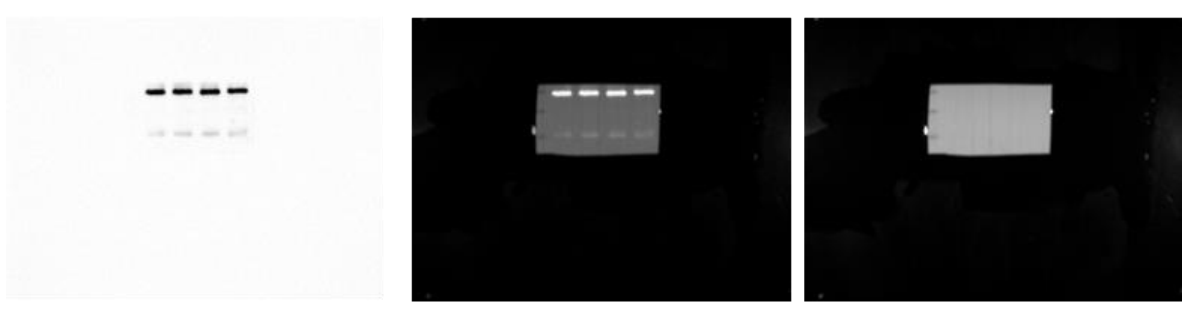

SW 480
β -actin
Figure2A
And
Figure3A

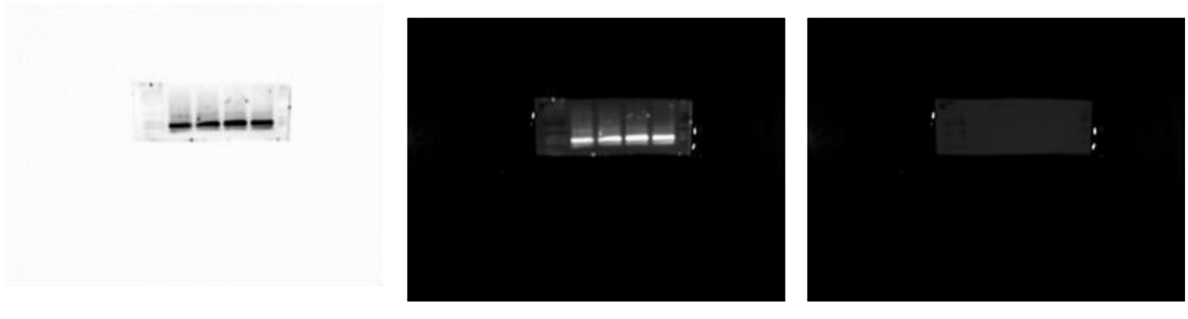

SW 480
P-STAT3
replicates

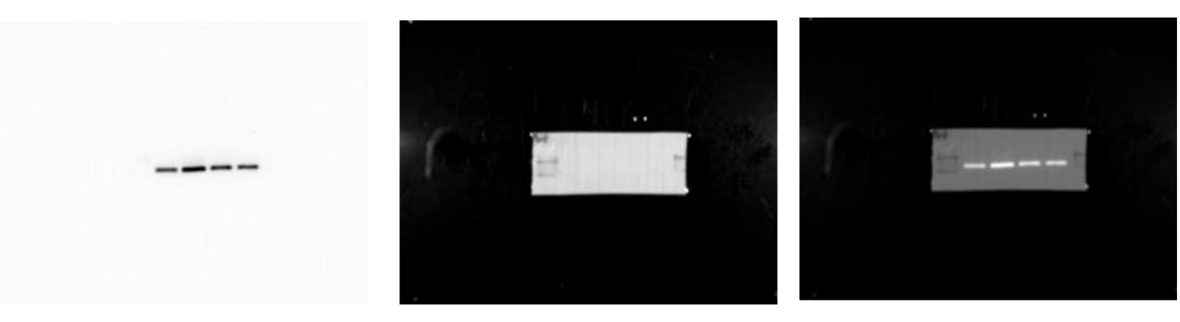

SW 480
P-STAT3
Figure2
and
Figure3A

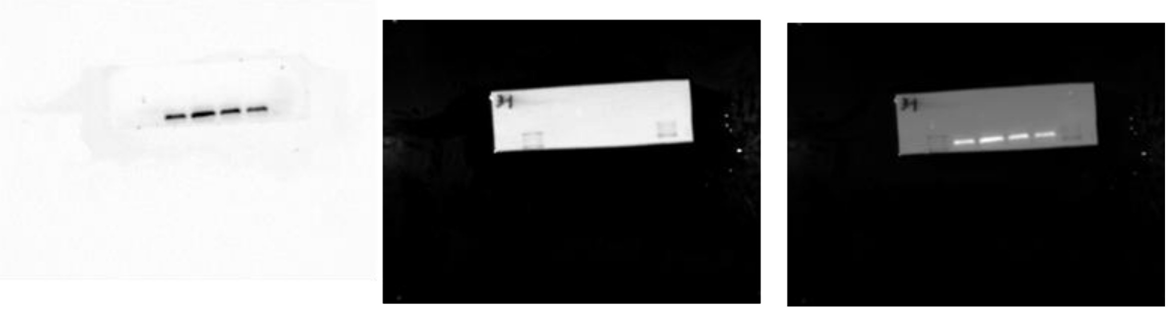

SW 480
P-STAT3
replicates

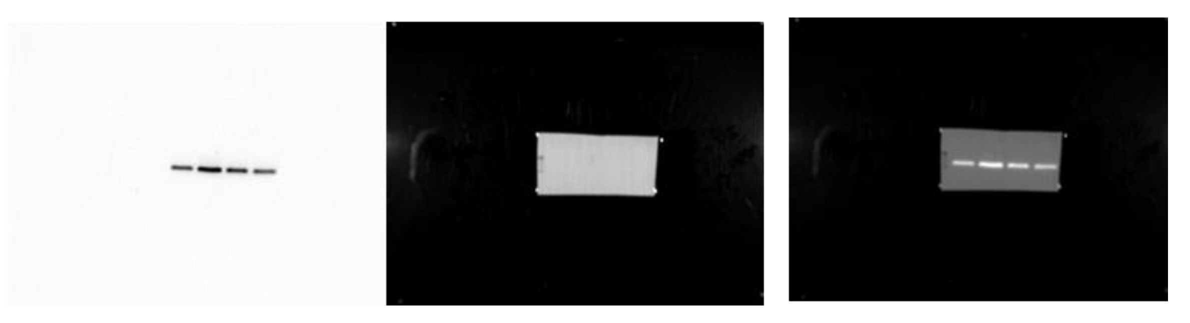

SW 480 STAT3
replicates

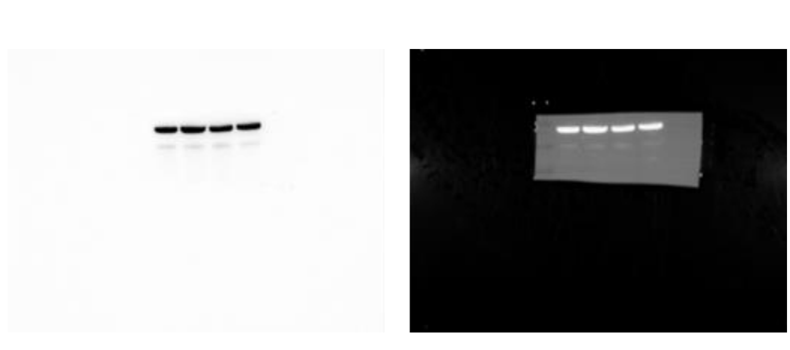

SW 480 STAT3
Figure2 A and
Figure3A

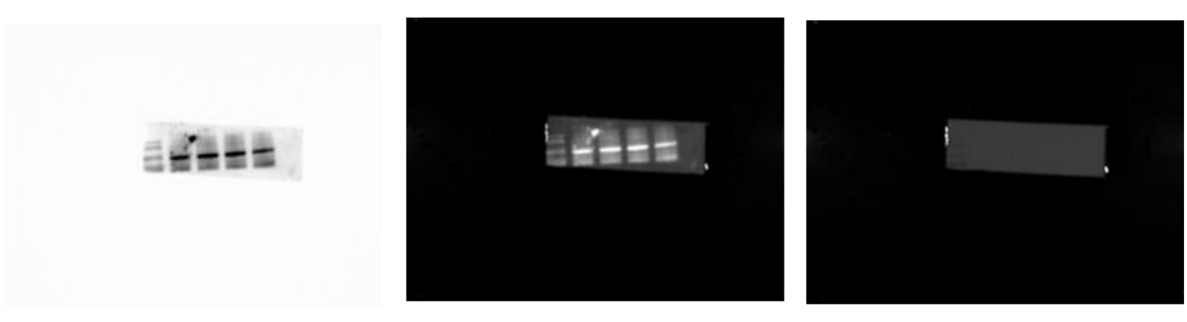

SW 480 STAT3

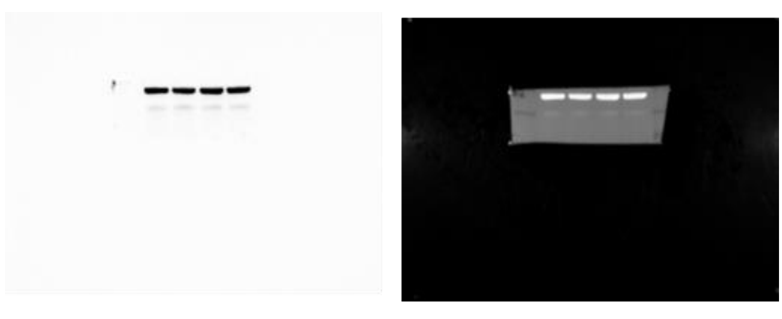

SW 480 slug
replicates

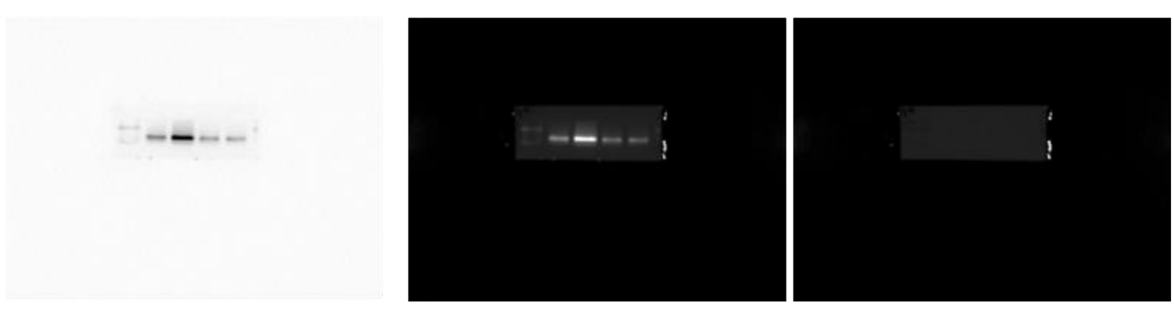

SW 480 slug
Figure4A

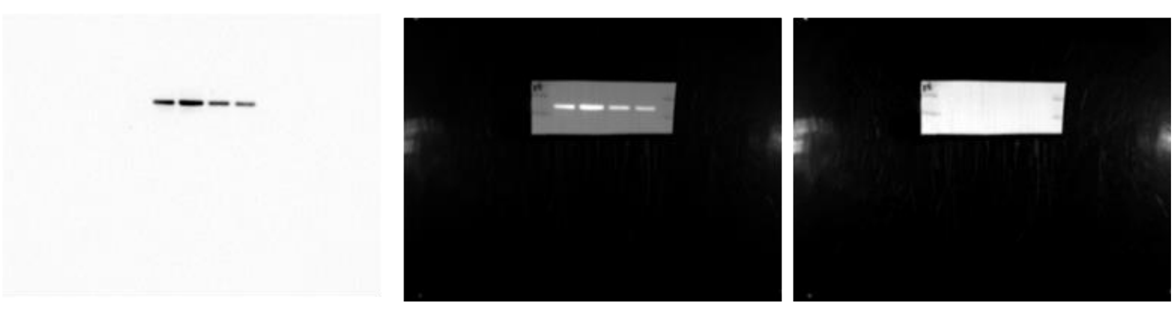

SW 480 slug
replicates

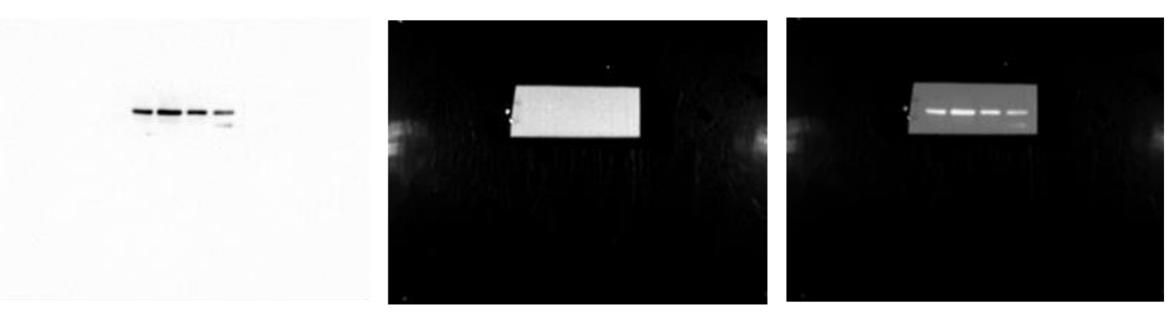

SW 480
E-cadherin
Figure4A

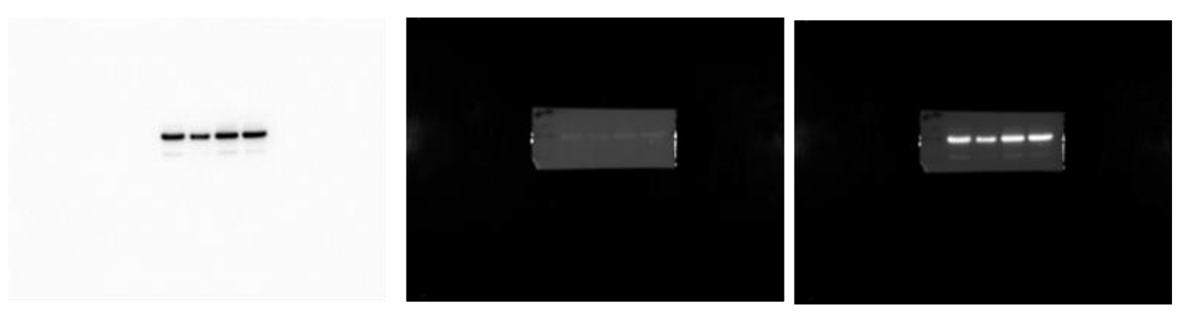

SW 480 E-cadherin
replicates

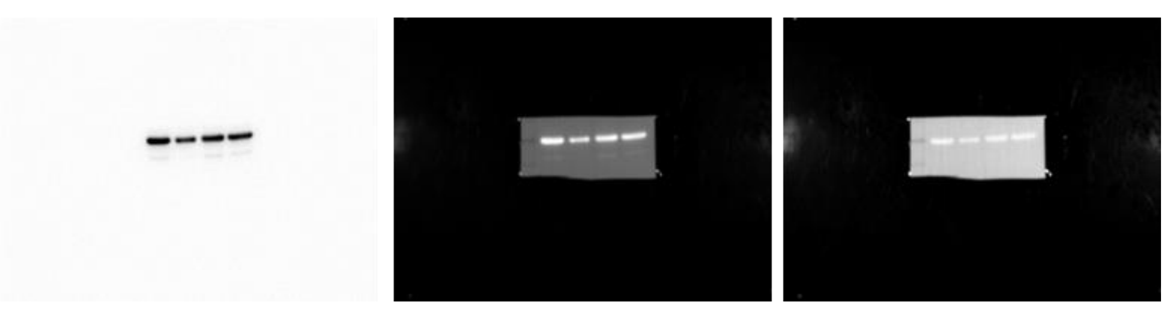

SW 480 E-cadherin
replicates

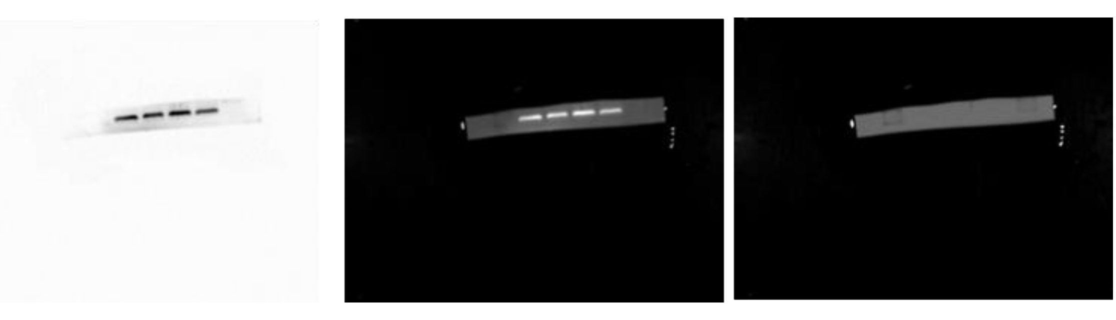

SW 480 Vimentin
Figure4A

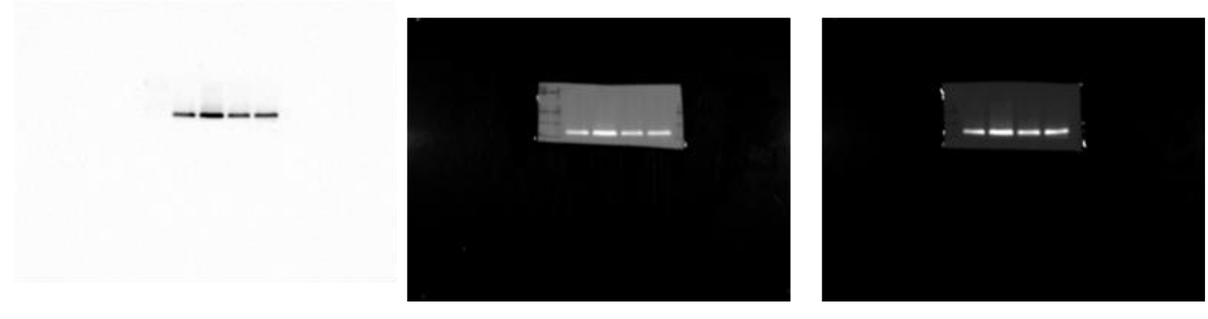

SW 480 Vimentin
replicates

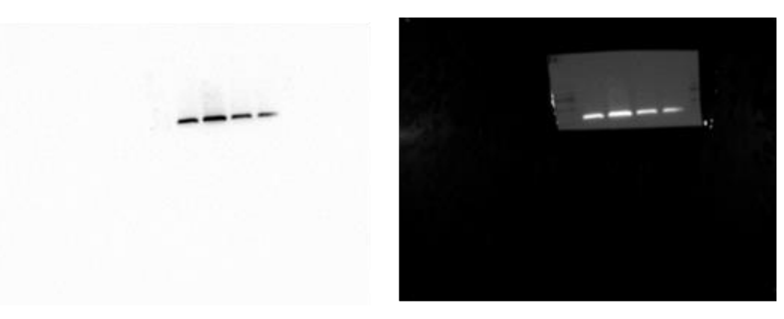

SW 480 Vimentin
replicates

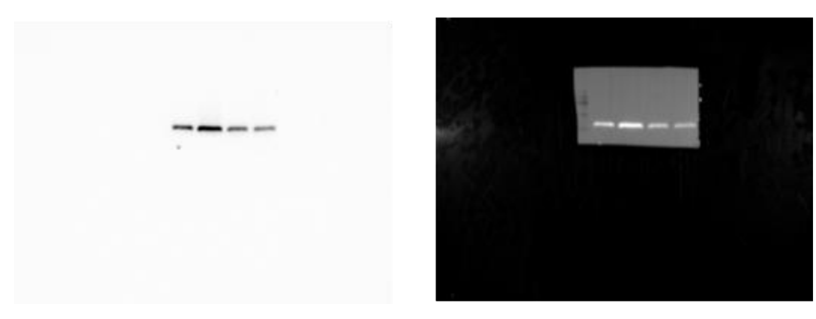

SW 480
N-cadherin
replicates

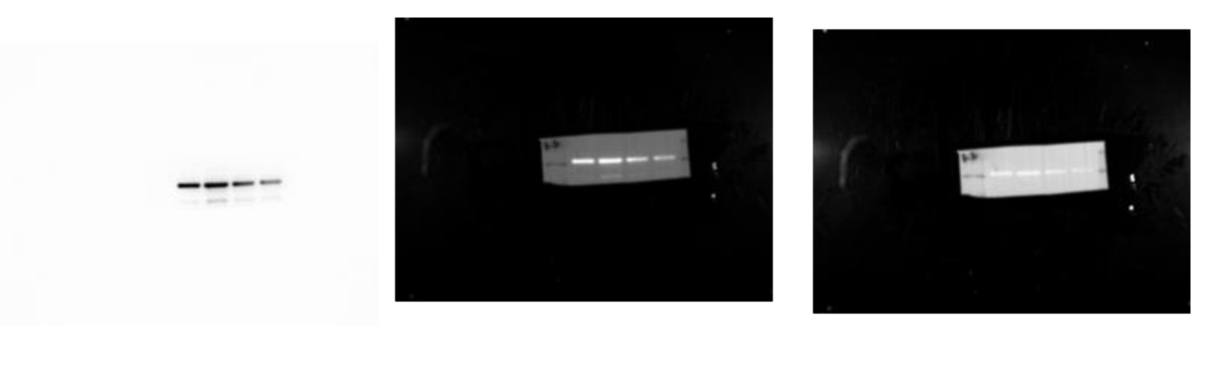

SW 480 N-cadherin
Figure4A

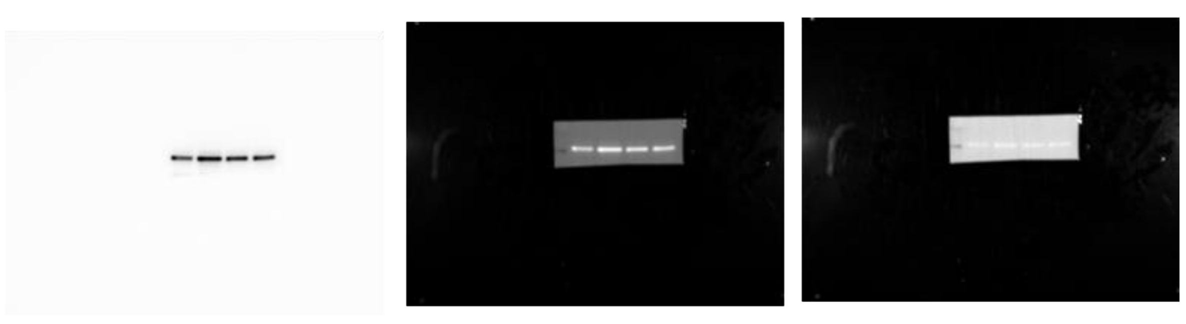

SW 480
N-cadherin
replicates

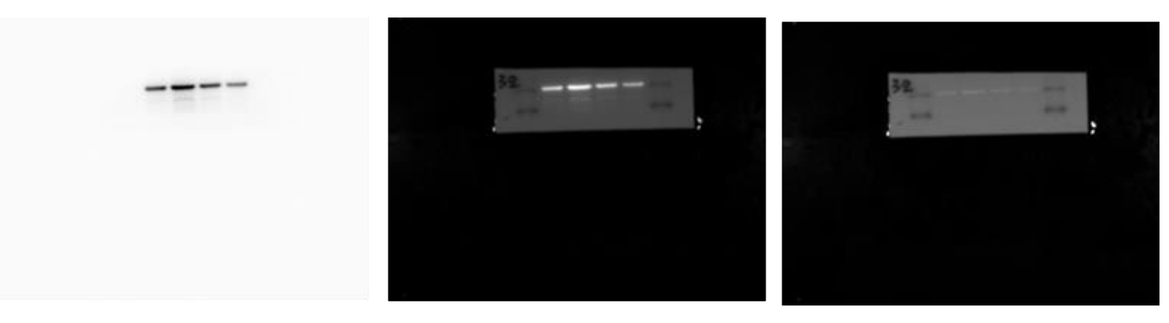

Supplement: Supplementary file 2 — Supplementary Material 2 [file 12876_2024_3195_MOESM2_ESM.pdf]
