# Supplementary material for: Effect and potential mechanism of oncometabolite succinate promotes distant metastasis of colorectal cancer by activating STAT3
Source: BMC Gastroenterol. 2024 Mar 14;24:106. doi: 10.1186/s12876-024-03195-x (PMC10938789; doi:10.1186/s12876-024-03195-x)

Nude mice-Western blot
Since some of the samples were in close proximity to the gel and the urgency of time, we had to crop the bands, and we guarantee that the samples derive from the same experiment and that gels/blots were processed in parallel.The blots were cut prior to hybridisation with antibodies during blotting,Because of time and experimental condition issues we did not have fuller-length

β-actin, Figure 5-C

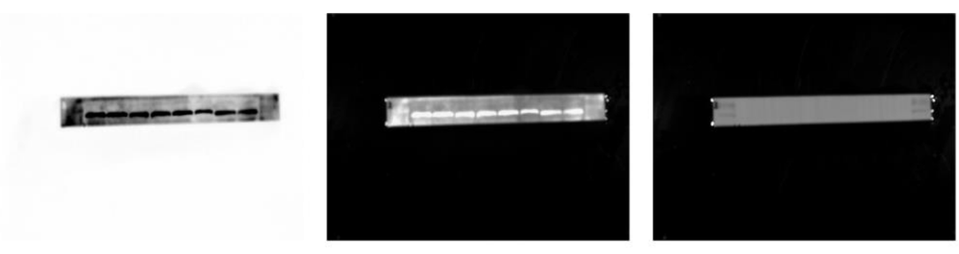

β-actin, replicates

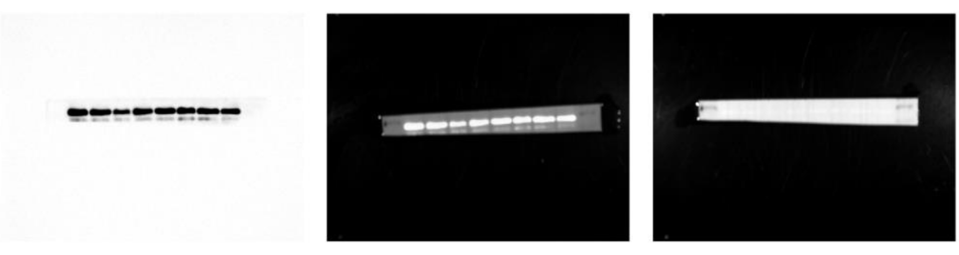

β-actin, Figure 5-E

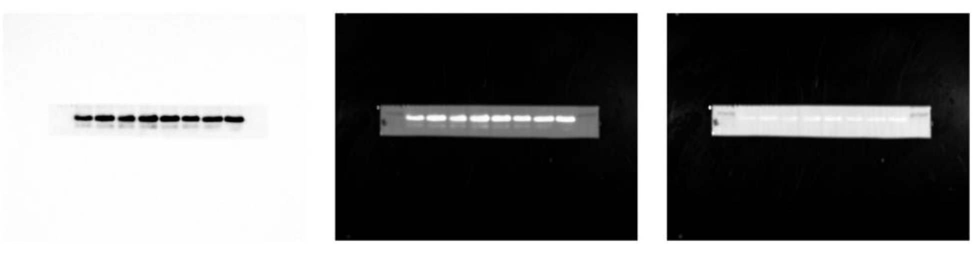

STAT3, Figure 5-C

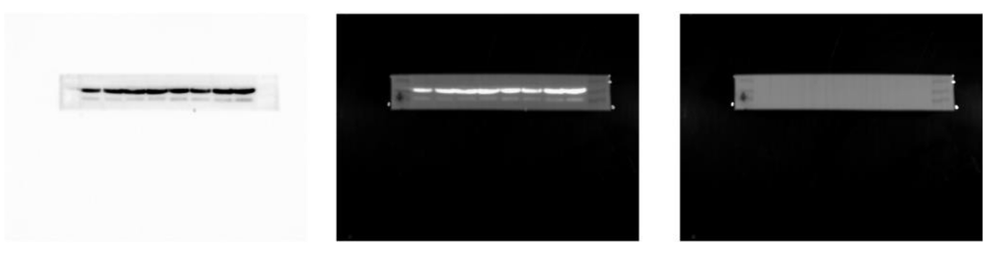

STAT3, replicates-1

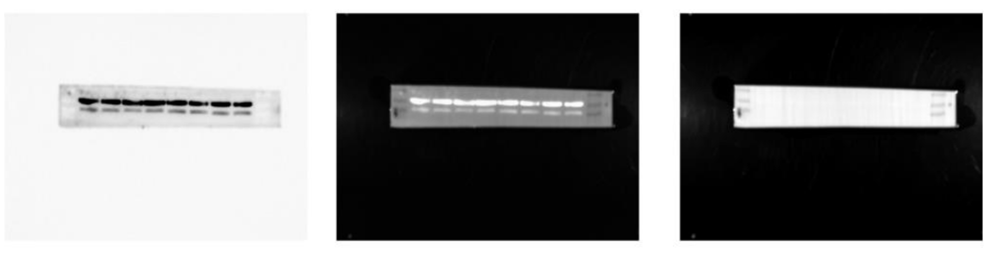

STAT3, replicates-2

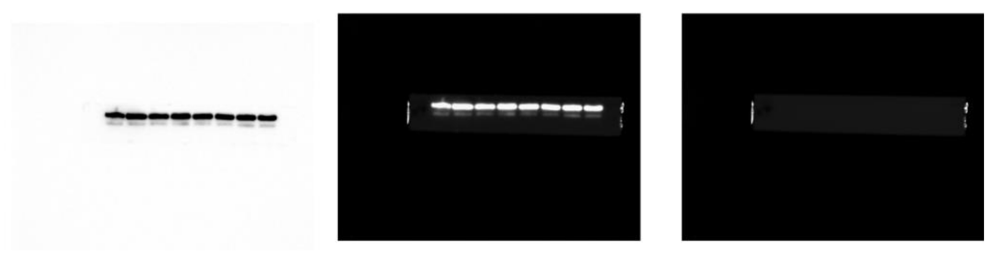

p-STAT3, Figure 5-C

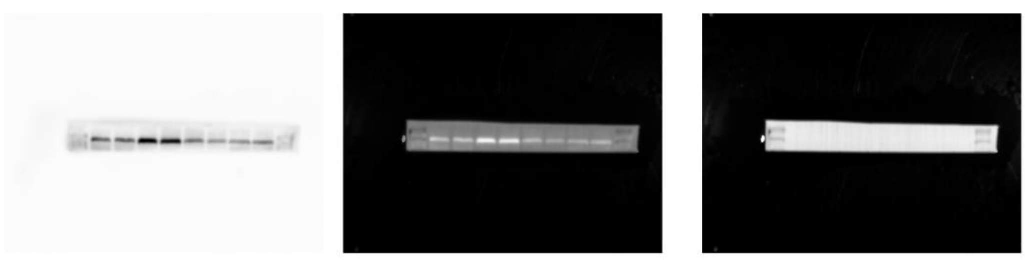

p- STAT3, replicates-1

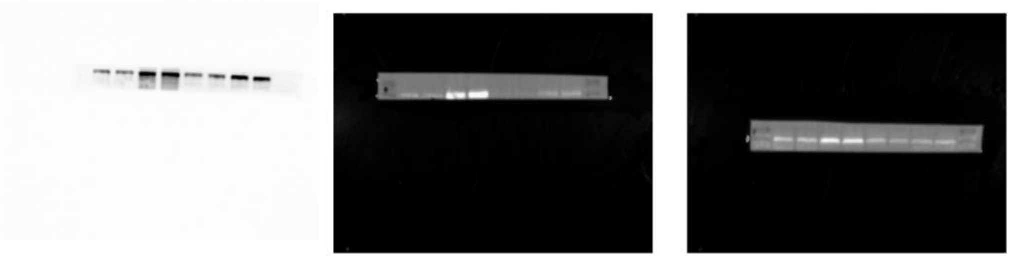

p-STAT3, replicates-2

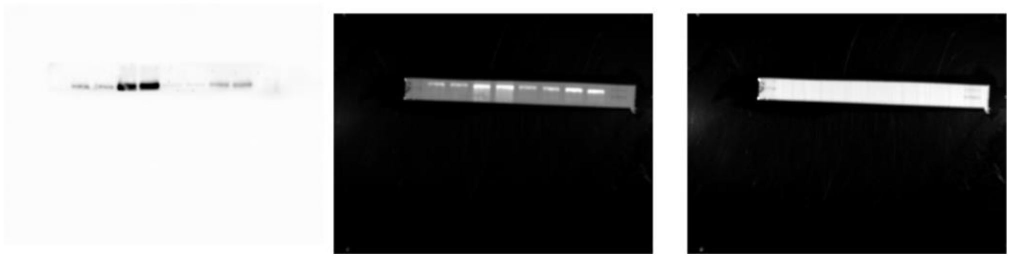

E-cadherin, Figure 5-E

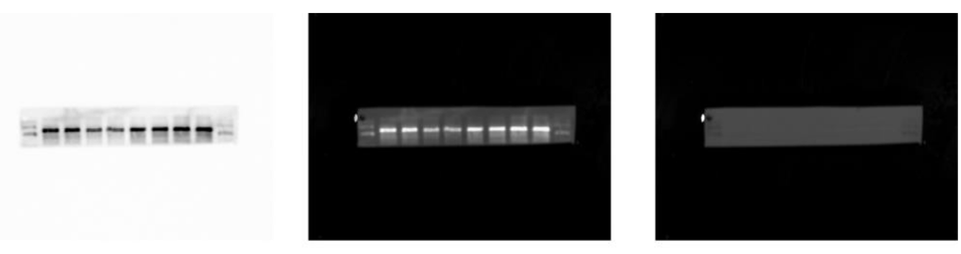

E-cadherin，replicates-1

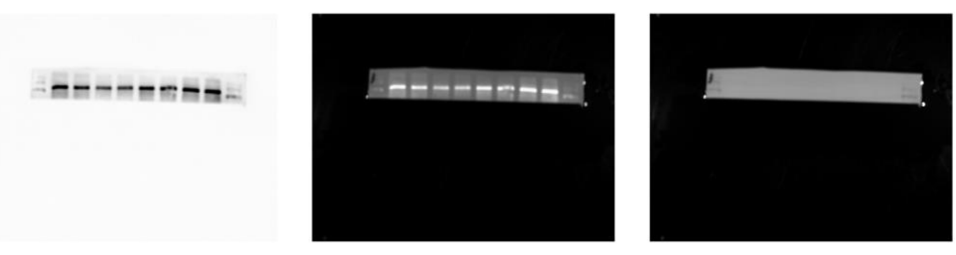

E-cadherin， replicates-2

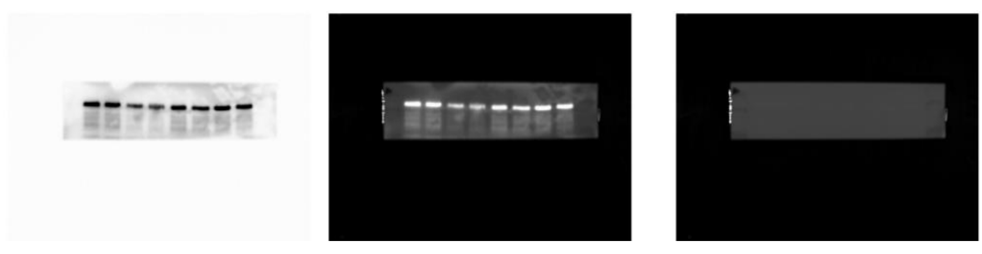

N-cadherin, Figure 5-E

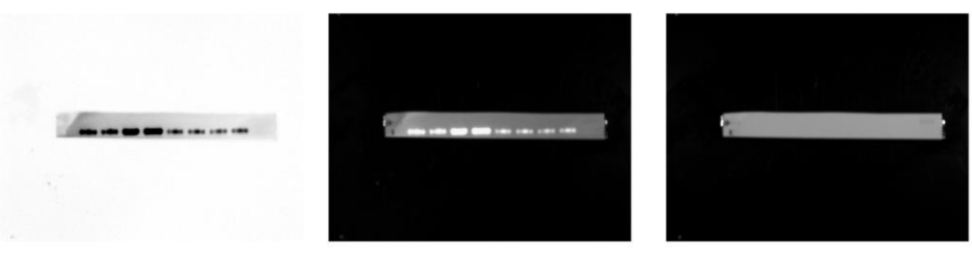

N-cadherin，replicates-1

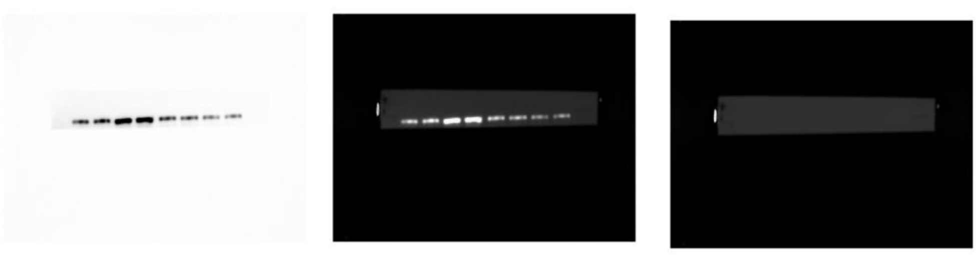

N-cadherin， replicates-2

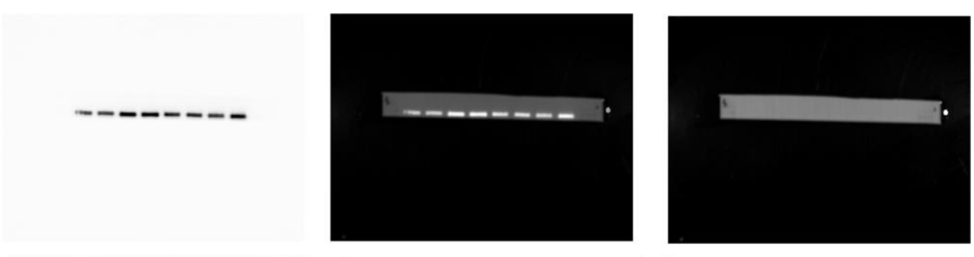

Slug, Figure 5-E

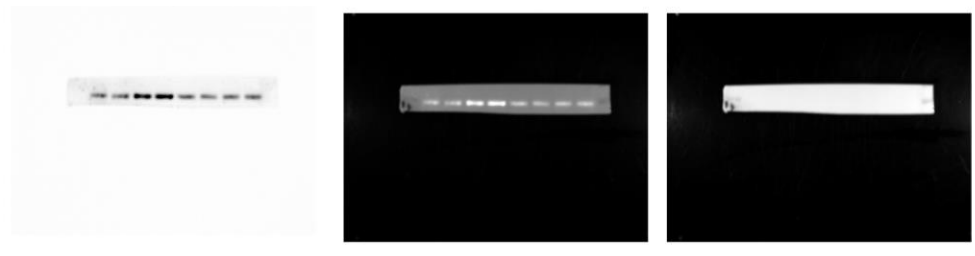

slug，replicates-1

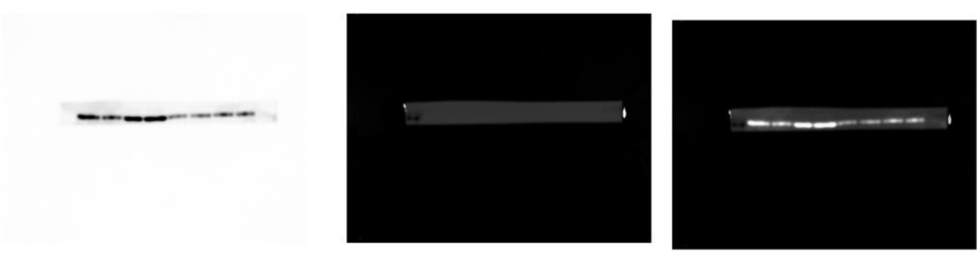

slug， replicates-2

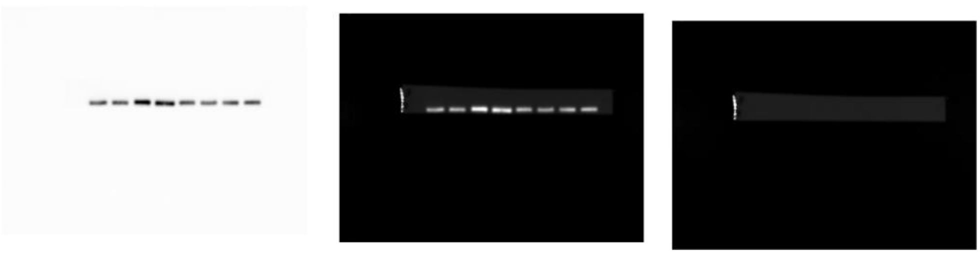

Vimentin, Figure 5-E

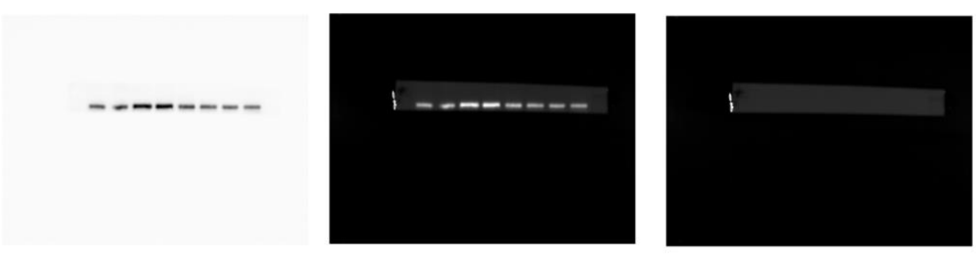

Vimentin，replicates-1

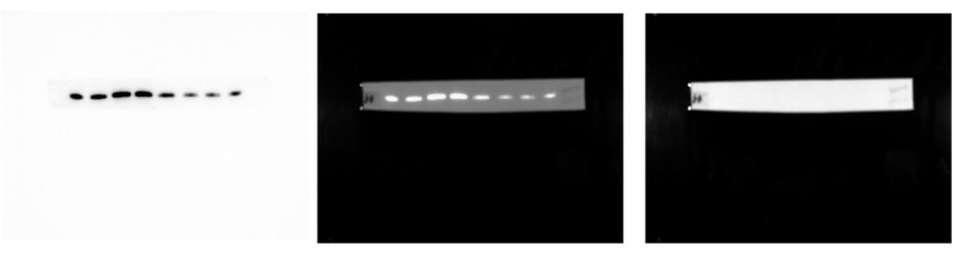

Vimentin， replicates-2

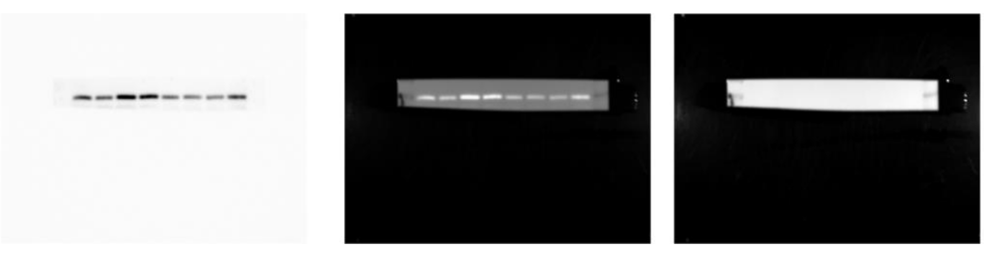

Supplement: Supplementary file 3 — Supplementary Material 3 [file 12876_2024_3195_MOESM3_ESM.pdf]
